# Supplementary material for: Enzymatic Fluoromethylation as a Tool for ATP‐Independent Ligation
Source: Angew Chem Int Ed Engl. 2023 Nov 29;63(1):e202312104. doi: 10.1002/anie.202312104 (PMC10952888; doi:10.1002/anie.202312104)
Supplement: Supplementary file 1 — Supporting Information [file ANIE-63-0-s001.pdf]

Angewandte Chemie
Eine Zeitschrift der Gesellschaft Deutscher Chemiker

Supporting Information

Enzymatic Fluoromethylation as a Tool for ATP-Independent Ligation

J. Peng, G. R. Hughes, M. M. Müller, F. P. Seebeck**

Methods and Material

Purchase of materials. Fluoromethyl iodide (FMeI) was purchased from abcr. *S*-adenosyl-L-methionine (SAM) and Tris(2-carboxyethyl)phosphine hydrochloride (TCEP.HCl) were purchased from Combi-Blocks. The following chemicals were purchased from Sigma Aldrich: isopropyl malic acid, iodomethane, *S*-adenosyl-L-homocysteine (SAH), coenzyme A disodium salt, indole-3-acetic acid, *O*-(4-nitrobenzyl) hydroxylamine, biotin-dPEG®$_3$-oxyamine HCl, 6-aminopenicillamine, *N,N'*-dimethylformamide (DMF), hydroxylamine solution. L-cysteine.HCl was purchased from ROTH. Hydrazine was purchased from fluorochem. 4-bromomethyl-7-methoxy-coumarin, chloroacetic anhydride was purchased from Acros. Sodium phosphate dibasic, sodium phosphate monobasic and sodium chloride were purchased from Thermo Scientific.

Nuclear Magnetic Resonance (NMR) measurement. NMR spectra were recorded on a Bruker 500 MHz instrument in described solvents. Data for are reported in chemical shift (δ ppm), multiplicity (s = singlet, d = doublet, dd = doublet of doublets, t = triplet, q = quartet, p = pentet, m = multiplet), coupling constant (Hz), integration.

High-resolution electron spray ionization mass spectrometry (HR-ESI-MS) measurement and analysis of protein samples. The mass spectrometer used was Bruker Maxis II paired with Shimadzu Nexera X2 LC series. Each protein sample was separated in LC column (Phenomenex C4) under buffer system A (0.01% formic acid in deionized water) and B (0.01% formic acid in acetonitrile). The LC program is as follow: 0-2 min, 10% B; 9-11 min, 55% B, 13-15 min, 90% B; 17-25 min, 10% B. At the beginning of each measurement, a standard calibrant was injected and recorded (50:50 isopropanol: H_2O, 10 mM NaOH, 53 mM formic acid). The ion polarity was set to positive mode, nebulizer at 1.8 bar. The flow rate of dry nitrogen gas was 8.0 L/min under a dry heater of 220 °C. 10.0 eV of collision energy and 4.0 eV of ionization energy were applied. The set capillary was at 4500 V. The set end plate offset was 500 V. The range of m/z from 300 to 3000 was scanned. Prior to analysis, each measured sample was internally calibrated using the reference list of sodium formate. The total ion count chromatogram was averaged to give m/z spectrum, treated by spectrum smoothing (0.8) and baseline subtraction (0.8). The protein molecular weight was calculated by applying maximal entropy deconvolution. The ion count intensity and molecular weight of the spectrum were exported as a simple XY file. In Excel 2016, the maximal ion count intensity in the XY file was set to 100% and the rest of ion count intensities were calculated corresponding to the maximum value. All ion count intensity was visualized as relative intensity. The relative intensity was plotted against molecular weight in OriginPro2021. Predicted spectra were generated following the formula of $(M + zH) \div z$. M is the molecular weight of the analyte, z is the charge of the protein. The error threshold for the deconvoluted protein mass is 50 ppm.

High-resolution electron spray ionization mass spectrometry (HR-ESI-MS) measurement and identifying of small molecules. The mass spectrometer used was Bruker Maxis II paired with Shimadzu Nexera X2 LC series. Each protein sample was separated in LC column (Gemini NX 5u C18, 100 × 4.6 mm) under buffer system A (0.01% formic acid in deionized water) and B (0.01% formic acid in acetonitrile). The LC concentration gradient was set as follow: 0-10 min 0% B, 15-20 min 100% B, 22-30 min 0% B. The first 6 min eluent was measured in MS. Scan range was from 50 m/z to 2000 m/z. Capillary was set at 4000 V, End Plate Offset at 500 V, Nebulizer at 1.8 bar, dry heater 220 °C. Dry N_2 at 8 L/min. At the end of each measurement, sodium formate solution was injected as an internal calibrant. Each measurement was calibrated according to the reference list of sodium formate. The small molecule of interest was identified by searching for the m/z value to four decimal places, giving the extracted ion chromatogram (EIC). The calculated m/z pattern was generated from the chemical formula of the small molecule in its protonated or deprotonated form with the corresponding charge. The presence of the small molecule of interest was indicated by a normal distribution curve of EIC, which was then averaged and aligned with the calculated EIC. The error threshold is 5 ppm.

Protein Sequences

>BxHMT (PDB 8AJP)
MGHHHHHHAENLYFQGSGSDPTQPAVPDFETRDPNSPAFWDERFERRFTPWDQAGVPAAFQSFAARHSGAAVLIPGCGSAYEAVWLAGQGNPVR
AIDFSPAAVAAAHEQLGAQHAQLVEQADFFTYEPPFTPAWIYERAFLCALPLARRADYAHRMADLLPGGALLAGFFFLGATPKGPPFGIERAEL
DALLTPYFDLIEDEAVHDSIAVFAGRERWLTWRRRA

>LahSB (PDB 6UAK)
MGHHHHHHAENLYFQGSGMEKEIKKWSVYFQNPEFLERTRMFLIQKELYPLVRNWCGVKDNVRLLDVGCGTGYFTRLLVSGDEDVSAVGIDMEE
PFIEYAREKAEELGLPAEFIIGDALALPFEDNTFDIVTSHTFLTSVPDPEKAMSEMKRVVKPGGIISSVTAMNFMPACNNEGEYPEECTWVEDL
KKEYMKIYTKYFSADPLETRIKGVKCSDVPKFFTGQGLKDVSLYPIGKVFTLSNAAVSDEDKLRYIELFYASEIKKLDAFMELPDEDIGITEED
AERFRSLIGQKCKWLRDHLHDNYAWEWQGGANLLVTGICNKQR

>TAMT (PDB 3G5T)
MSSTFSASDFNSERYSSSRPSYPSDFYKMIDEYHDGERKLLVDVGCGPGTATLQMAQELKPFEQIIGSDLSATMIKTAEVIKEGSPDTYKNVSF
KISSSDDFKFLGADSVDKQKIDMITAVECAHWFDFEKFQRSAYANLRKDGTIAIWGYADPIFPDYPEFDDLMIEVPYGKQGLGPYWEQPGRSRL
RNMLKDSHLDPELFHDIQVSYFCAEDVRDKVKLHQHTKKPLLIRKQVTLVEFADYVRTWSAYHQWKQDPKNKDKEDVADWFIKESLRRRPELST
NTKIEVVWNTFYKLGKRVLEHHHHHH

>IAAMT (PDB 3B5I)
MGHHHHHHAENLYFQGSGKLERLLSMKGGKGQDSYANNSLAQAMHARSMLHLLEETLENVHLNSSASPPPFTAVDLGCSSGANTVHIIDFIVKH
ISKRFDAAGIDPPEFTAFFSDLPSNDFNTLFQLLPPLVSNTCMEECLAADGNRSYFVAGVPGSFYRRLFPARTIDFFHSAFSLHWLSQVPESVT
DRRSAAYNRGRVFIHGAGEKTTTAYKRQFQADLAEFLRARAAEVKRGGAMFLVCLGRTSVDPTDQGGAGLLFGTHFQDAWDDLVREGLVAAEKR
DGFNIPVYAPSLQDFKEVVDANGSFAIDKLVVYKGGSPLVVNEPDDASEVGRAFASSCRSVAGVLVEAHIGEELSNKLFSRVESRATSHAKDVL
VNLQFFHIVASLSFT

>GFP-tag-1 (PDB 2B3P)
MGHHHHHHAENLYFQGSGMSKGEELFTGVVPILVELDGDVNGHKFSVRGEGEGDATNGKLTLKFICTTGKLPVPWPTLVTTLTYGVQCFSRYPD
HMKRHDFFKSAMPEGYVQERTISFKDDGTYKTRAEVKFEGDTLVNRIELKGIDFKEDGNILGHKLEYNFNSHNVYITADKQKNGIKANFKIRHN
VEDGSVQLADHYQQNTPIGDGPVLLPDNHYLSTQSVLSKDPNEKRDHMVLLEFVTAAGITHGMDELYKGSGDGDEVDYSLFAATAM

>GFP-tag-2 (PDB 2B3P)
MGHHHHHHAENLYFQGSGMSKGEELFTGVVPILVELDGDVNGHKFSVRGEGEGDATNGKLTLKFICTTGKLPVPWPTLVTTLTYGVQCFSRYPD
HMKRHDFFKSAMPEGYVQERTISFKDDGTYKTRAEVKFEGDTLVNRIELKGIDFKEDGNILGHKLEYNFNSHNVYITADKQKNGIKANFKIRHN
VEDGSVQLADHYQQNTPIGDGPVLLPDNHYLSTQSVLSKDPNEKRDHMVLLEFVTAAGITHGMDELYKGSGFLGSAIVAASSAGAV

>GFP-tag-3 (PDB 2B3P) 2B3P)
MGHHHHHHAENLYFQGSGMSKGEELFTGVVPILVELDGDVNGHKFSVRGEGEGDATNGKLTLKFICTTGKLPVPWPTLVTTLTYGVQCFSRYPD
HMKRHDFFKSAMPEGYVQERTISFKDDGTYKTRAEVKFEGDTLVNRIELKGIDFKEDGNILGHKLEYNFNSHNVYITADKQKNGIKANFKIRHN
VEDGSVQLADHYQQNTPIGDGPVLLPDNHYLSTQSVLSKDPNEKRDHMVLLEFVTAAGITHGMDELYKGSGDGDEVDYSLFAATAI

>Nanobody (PDB 3OGO)
MGSSHHHHHHSSGLVPRGSHMQVQLVESGGALVQPGGSLRLSCAASGFPVNRYSMRWYRQAPGKEREWVAGMSSAGDRSSYEDSVKGRFTISRD
DARNTVYLQMNSLKPEDTAVYYCNVNVGFEYWGQGTQVTVSSGSGDGDEVDYSLFAATAM

>Nanobody-C16 (PDB 3OGO)
MGHHHHHHAENLYFQCSGSQVQLVESGGALVQPGGSLRLSCAASGFPVNRYSMRWYRQAPGKEREWVAGMSSAGDRSSYEDSVKGRFTISRDDA
RNTVYLQMNSLKPEDTAVYYCNVNVGFEYWGQGTQVTVSS

>FalD (PDB 1KOL)
MGHHHHHHAENLYFQGSGGNRGVVYLGSGKVEVQKIDYPKMQDPRGKKIEHGVILKVVSTNICGSDQHMVRGRTTAQVGLVLGHEITGEVIEKG
RDVENLQIGDLVSVPFNVACGRCRSCKEMHTGVCLTVNPARAGGAYGYVDMGDWTGGQAEYVLVPYADFNLLKLPDRDKAMEKIRDLTCLSDIL
PTGYHGAVTAGVGPGSTVYVAGAGPVGLAAAASARLLGAAVVIVGDLNPARLAHAKAQGFEIADLSLDTPLHEQIAALLGEPEVDCAVDAVGFE
ARGHGHEGAKHEAPATVLNSLMQVTRVAGKIGIPGLYVTEDPGAVDAAAKIGSLSIRFGLGWAKSHSFHTGQTPVMKYNRALMQAIMWDRINIA
EVVGVQVISLDDAPRGYGEFDAGVPKKFVIDPHKTFSAA

>PIMT (PDB 1I1N)
MHHHHHHSSGLVPRGSGMKETAAAKFERQHMDSPDLGTDDDDKAMAWKSGGASHSELIHNLRKNGIIKTDKVFEVMLATDRSHYAKCNPYMDSP
QSIGFQATISAPHMHAYALELLFDQLHEGAKALDVGSGSGILTACFARMVGCTGKVIGIDHIKELVDDSVNNVRKDDPTLLSSGRVQLVVGDGR
MGYAEEAPYDAIHVGAAAPVVPQALIDQLKPGGRLILPVGPAGGNQMLEQYDKLQDGSIKMKPLMGVIYVPLTDKEKQWSRDEL

Protein Production and Purification

Transformation. pET28a-(+) encoded plasmids were transformed into SAH nucleosidase deficient *E.coli* strain (Δmtn)[1] by electroporation using BIO-RAD GENE PULSER® II. The volts was set to 2 kV and the resulting time constant was around 5 msec. Capacitance was set at 30 μF. Immediately after applying the electric pulse, 1 mL of 37 °C sterile LB medium was added to the cuvette and mixed with the cell culture. The cell culture was incubated at 37 °C for a minimum of 45 min before plating on LB-Agar plates (5 mL LB-agar with 50 μg/mL kanamycin).

Production of recombinant HMT, LahS$_B$, TAMT, IAAMT, Nanobody, Nanobody-C16, FalD. Colony containing the plasmid was collected from LB-AGAR plates and transferred to a pre-culture of 5 mL LB medium containing kanamycin 50 μg/ml. After incubation at 37 °C overnight, per liter of fresh Terrific Broth (TB) medium (1L) with Kanamycin (50 μg/ml) was inoculated by 5 mL of pre-culture. Each 1 L of cultures were incubated in 3 L shaking flask at 37 °C (180 rpm) until OD_{600} reached 0.6. Isopropyl *β*-D-thiogalactoside (IPTG) was added to the final concentration of 100 μM. The protein was allowed to express at 18 °C for 16 hours. Cells were harvested by centrifugation at 5,000 × g for 20 minutes at 4 °C and stored at -20 °C for a minimum of 2 hours before purification.

Production of recombinant GFP-tag-1,2,3. Colony containing the plasmid was collected from LB-AGAR plates and transferred to a pre-culture of 5 mL LB medium containing kanamycin 50 μg/ml. After incubation at 37 °C overnight, per liter of fresh Terrific Broth (TB) medium (1L) with Kanamycin (50 μg/ml) was inoculated by 5 mL of pre-culture. Each 1 L of cultures were incubated in 3 L shaking flask at 37 °C (180 rpm) until OD_{600} reached 0.6.[2] IPTG was added to the final concentration of 100 μM. The protein was allowed to express for 4 hours at 35 °C. Cells were harvested by centrifugation at 5,000 × g for 20 minutes at 4 °C and stored at -20 °C overnight before purification.

Protein purification. Frozen cell pellets were suspended in 4 mL/g_{cww} lysis buffer (50 mM phosphate, 100 mM NaCl, pH 8.0). The suspension was disrupted at 4 °C by sonication for 3 x 120 s with a Branson sonifier 450 (output control 5, 50% duty cycle). The suspension was homogenized at 4 °C by EmulsiFlex at 1000 bar applied with 4 bar pressure of N_2. Soluble fraction was collected after centrifugation at 10,000 x g for 45 min at 4 °C. Every 25 mL of clear lysate was mixed with 1 ml of Ni^{II} NTA agarose at 4 °C for 20 min and loaded onto a column (10 mL polypropylene column, Thermo Scientific) with a matching polyethylene disc at the bottom. The agarose beads were washed with 10 ml cold lysis buffer containing 10 mM and 20 mM imidazole respectively. The protein was eluted in 400 μL fractions with cold lysis buffer solution containing 250 mM imidazole. The absorbance at 280 nm of the eluted fractions was measured on NanoDrop 2000 Spectrophotometer (Thermo Scientific). Fractions with Abs_{280nm} > 0.2 were combined and dialyzed with a cellulose bag (MW 6-8 kDa cutoff, 28 μm thick, art.# E663.1, purchased from ZelluTransROTH®) against dialysis buffer (50 mM phosphate, pH 8). The final protein concentration was determined by measuring Abs_{280nm} against the dialysis buffer. The protein was aliquoted, frozen and stored at -80 °C. The enzymes purity was justified by SDS-PAGE. 1 μL of 0.2 mg/mL of protein was submitted for analysis by HR-ESI-MS.

N-terminal His-tag removal of Nanobody-C16. After dialysis, TEV protease was added to the protein sample and incubated at 25 °C for 2 hours. The ratio between protein and TEV protease was 50:1. The mixture was loaded onto pre-equilibrated Ni-NTA column. The eluent from the flow-through was collected. The column was washed with 10, 20 and 250 mM imidazole buffer. An SDS-PAGE gel was run to check the homogeneity of the eluent. The homogeneous fraction of lower molecular weight was combined and dialyzed against 50 mM sodium phosphate buffer, pH 8.0. The protein was concentrated to more than 2 mM, then aliquoted, flash frozen and stored at -80 °C.

Isopropyl Malic Acid and Cysteine

Isopropyl malic acid was the first example to demonstrate using FMeI to activate the carboxyl group. Amide bond was formed between cysteine and isopropyl malic acid (**1**) following the principle of native chemical ligation.

Analytical Scale. In a 200 µL reaction in 50 mM sodium phosphate buffer pH 8.0 at 25 °C, 1 mM of isopropyl malic acid was incubated with 4 mM FMeI, 40 µM of SAH, 10 µM of HMT, 10 µM of TAMT, 4 mM cysteine, 4 mM TCEP.HCl in 50 mM sodium phosphate buffer, pH 8.0 at 25 °C.[3] After 19 hour of reaction, 1 µL of the reaction mixture was diluted to 50 µL and centrifuged. The formation of compound **4** was confirmed by HRMS. Compound **4** $[C_{10}H_{16}NO_6S]^-$ calculated 278.0703, found 278.0707. The starting material IPMA (**1**, $[C_7H_{11}O_5]^-$, 175.0611) was not detected in extracted ion count. In the control reaction with MeI instead of FMeI, **1** was methylated to produce **2b**.

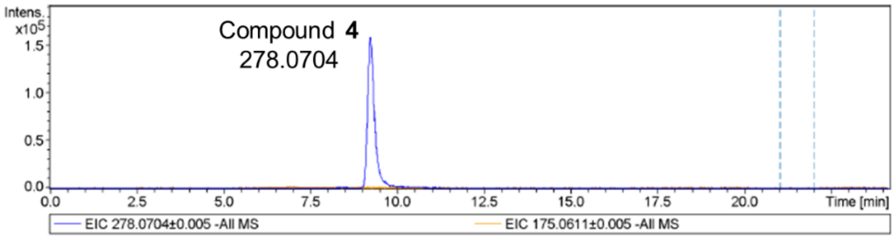

Figure S1. EIC chromatogram confirming the formation of compound **4**. Extracted ion count (EIC) chromatogram of compound **4** (278.0704 ± 0.005) and **1** (175.0611 ± 0.005) extracted from the measurement of the reaction mixture after 19 hours.

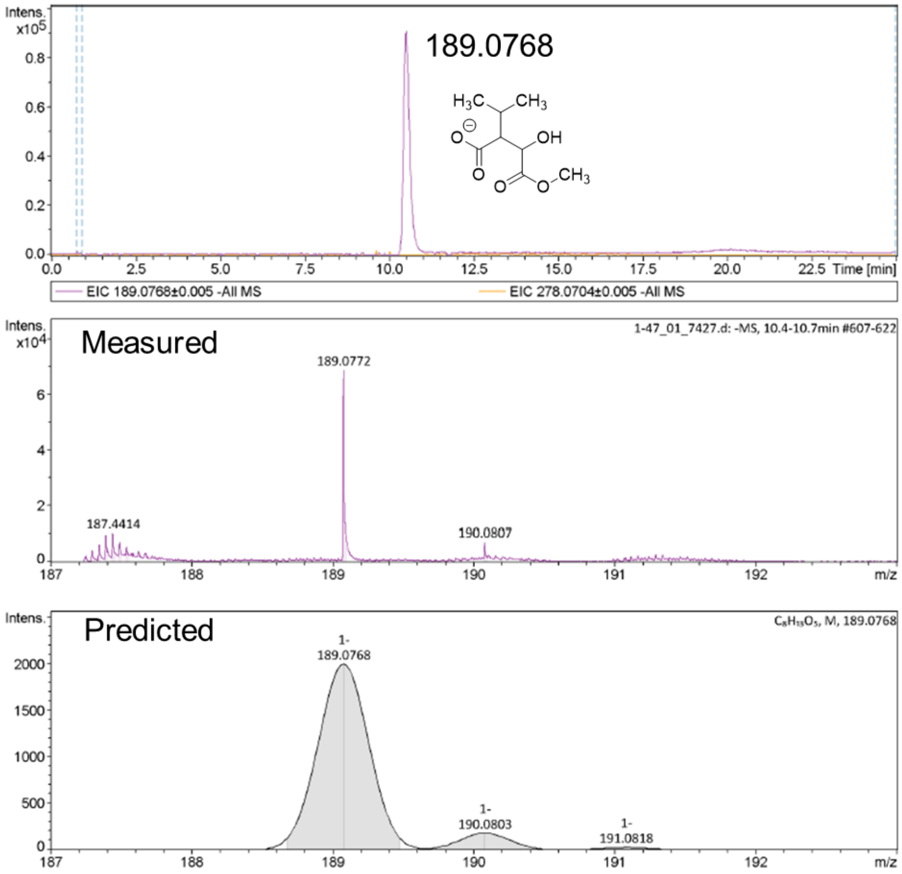

Figure S2. EIC chromatogram confirming the formation of **2b** (189.0768 ± 0.005).

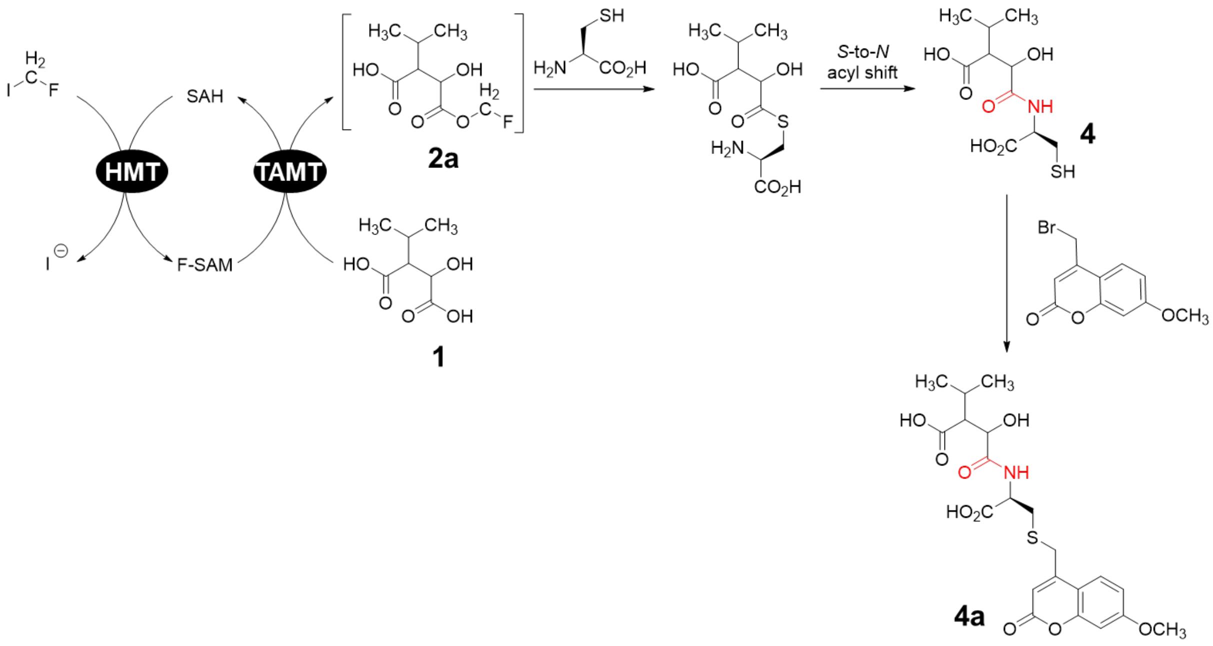

Figure S3. HMT-MT cascade for the biosynthesis of compound **4** and **4a**. Compound **1** is activated by F-methylation and the incubation with cysteine leads to the formation of amide bond via native chemical ligation. 4-bromomethyl-7-methoxy-coumarin is added to confirm the *S* to *N* shift and to serve as a chromophore for product detection and isolation via HPLC-UV.

Production and characterization of 4a. Isopropyl malic acid (**1**, 11.5 mg, 0.064 mmol), L-cysteine (17.2 mg, 0.108 mmol) was stirred to mix in 6.9 mL of 50 mM sodium phosphate buffer (pH 8.0) in a 50 mL round bottom flask. 50 µM of SAH was added to the mixture. The mixture was degassed through three cycles of freeze-pump-thaw. 50 µM HMT and 50 µM TAMT were mixed and added to the degassed reaction mixture, followed by the addition of FMeI (60 µL, 0.16 mmol). The 10 mL reaction was stirred at 22 °C overnight under constant flow of N_2 in Schlenk line. The reaction was monitored by ESI-MS paired with LC. After the majority of compound **1** was consumed (by HR-ESI-MS, Figure 4), 4-bromomethyl-7-methoxy-coumarin in acetonitrile (40 mg, 0.15 mmol) was degassed and added to the reaction. The white suspension was stirred at 45 °C overnight under constant flow of N_2. The mixture became clear after overnight stirring. The reaction mixture was reduced under vacuum at 45 °C after 60 min of centrifuge using SpeedVac (Eppendorf Concentrator plus). Enzymes and salts remained in the precipitant. The supernatant was purified using semi-preparative HPLC (ThermoScientific, Chromeleon) paired with a reverse phase column (Gemini-NX 5u C18 110 Å, 250 × 4.6 mm) and UV-Vis detector. The HPLC program was run under buffer A (97% deionized H_2O, 3% MeCN, 0.1% TFA) and buffer B (0.1% TFA in MeCN). The buffer gradient was 5% buffer B (0 - 1 min), 95% buffer B (7 - 11.2 min), 5% buffer B (11.4 - 15.6 min). The absorption at 300 nm was monitored. The fraction from 9.8 to 10.1 min was collected. The solvent was removed by rotatory evaporation and lyophilization. The isolated compound **4a** appeared as a light white powder (24 mg, 0.050 mmol, 78% yield). 480 µL of CD_3CN was added to the white powder and NMR was recorded.

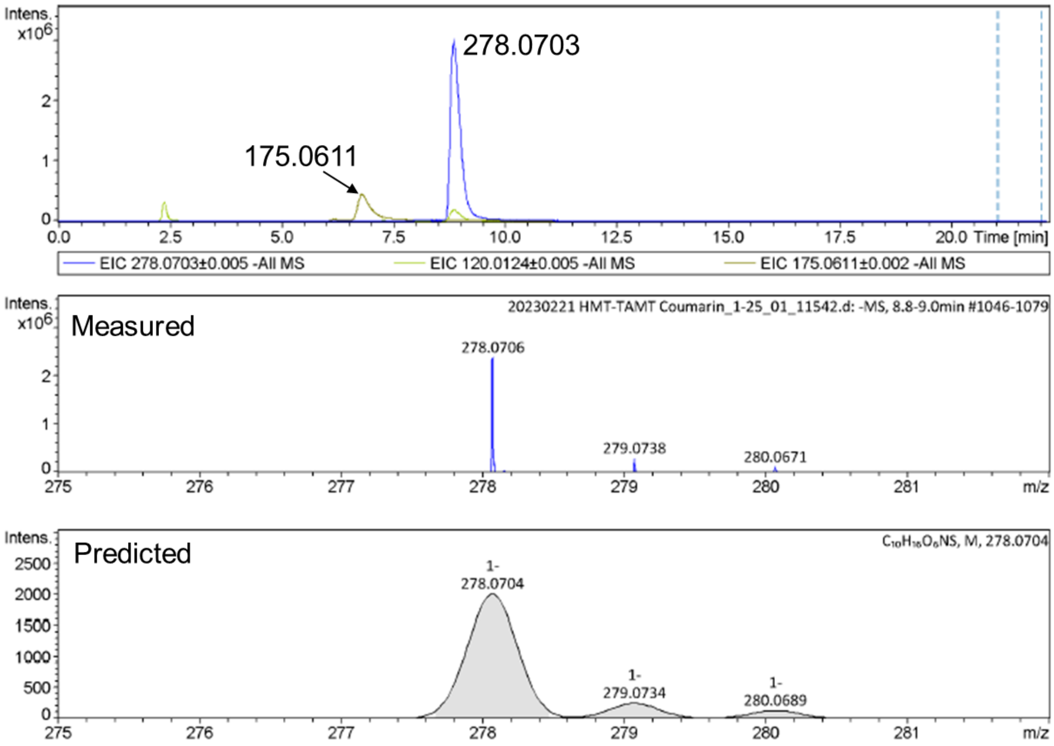

Figure S4. HR-ESI-MS spectrum confirming the formation of compound **4** prior to alkylation with 4-bromomethyl-7-methoxy-coumarin. **Top:** EIC chromatogram of compound **4** (278.0704 ± 0.005) and **1** (175.0611 ± 0.005) extracted from the measurement of the reaction mixture after 18 hours. **Middle:** averaged spectrum from the EIC chromatogram, measured m/z of $[C_{10}H_{16}NO_6S]^-$ is 278.0706. Bottom: calculated m/z pattern of compound **4**, $[C_{10}H_{16}NO_6S]^-$, 278.0704.

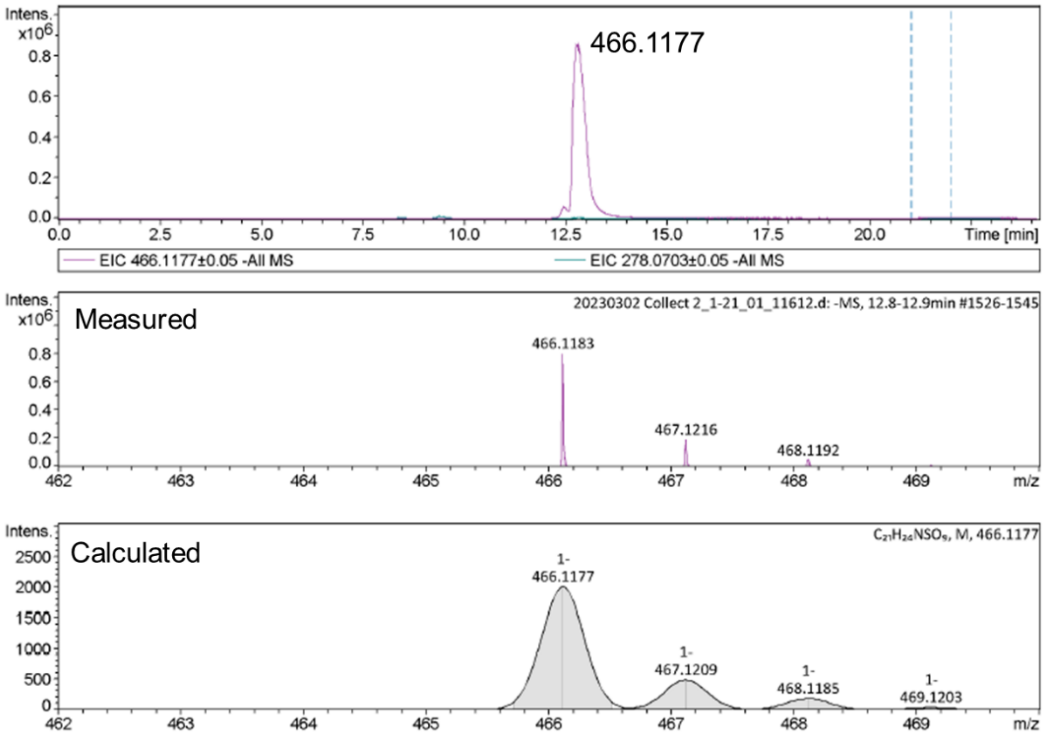

Figure S5. HR-ESI-MS spectrum confirming the formation and isolation of compound **4a. Top:** EIC chromatogram of compound **4a** (466.1177 ± 0.005) purified after semi-preparative column. **Middle:** averaged spectrum from the EIC chromatogram, measured m/z of $[C_{21}H_{22}NO_9S]^-$ is 466.1183. **Bottom:** calculated m/z pattern of compound **4a**,$[C_{21}H_{22}NO_9S]^-$, 466.1177.

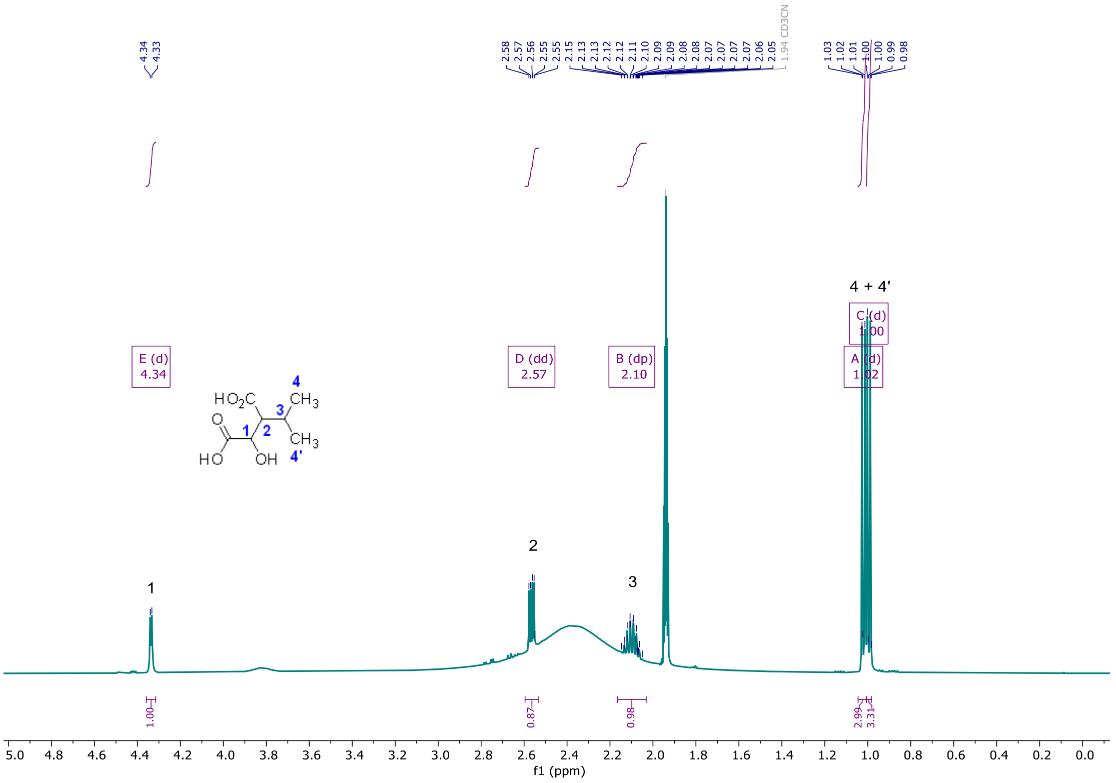

Figure S6. ^{1}H NMR of commercial **1.** (500 MHz, CD_3CN) δ 4.34 (d, *J* = 3.9 Hz, 1H), 2.57 (dd, *J* = 8.1, 3.9 Hz, 1H), 2.10 (dp, *J* = 8.1, 6.7 Hz, 1H), 1.02 (d, *J* = 6.8 Hz, 3H), 1.00 (d, *J* = 6.8 Hz, 3H). The manufacturer gave no indication of stereochemistry. The following structure of the product was therefore drawn without indication of stereochemistry.

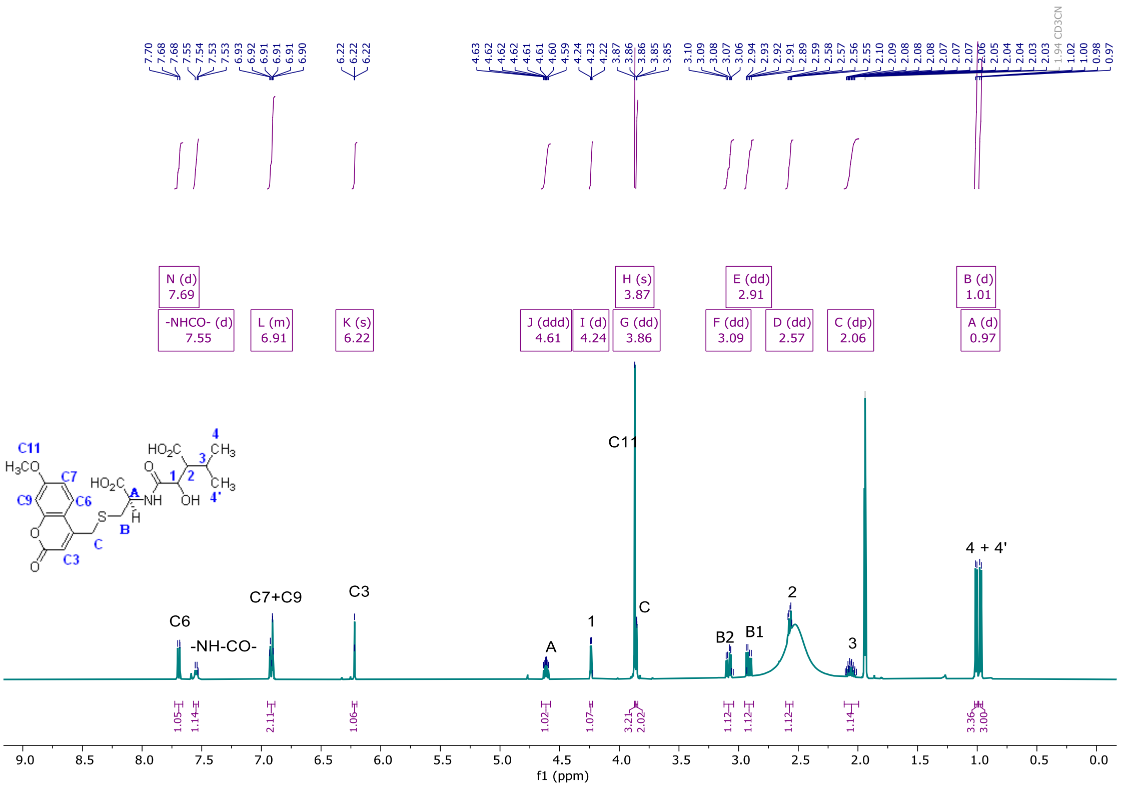

Figure S7. ^{1}H NMR of Compound **4a**. (500 MHz, CD_3CN). δ 7.69 (d, *J* = 8.5 Hz, 1H), 7.55 (d, *J* = 8.0 Hz, 1H), 6.91 (m, 2H), 6.22 (d, *J* = 0.9 Hz, 1H), 4.61 (ddd, *J* = 8.0, 7.2, 5.0 Hz, 1H), 4.24 (d, *J* = 3.7 Hz, 1H), 3.87 (s, 3H), 3.86 (dd, *J* = 2.2, 0.8 Hz, 2H), 3.09 (dd, *J* = 14.1, 5.1 Hz, 1H), 2.91 (dd, *J* = 14.1, 7.2 Hz, 1H), 2.57 (dd, *J* = 8.5, 3.6 Hz, 1H), 2.06 (dp, *J* = 8.5, 6.8 Hz, 1H), 1.01 (d, *J* = 6.7 Hz, 3H), 0.97 (d, *J* = 6.7 Hz, 3H). The broad peak at 2.5 ppm is water, coming from H-D exchange of –CO_2H and –OH.

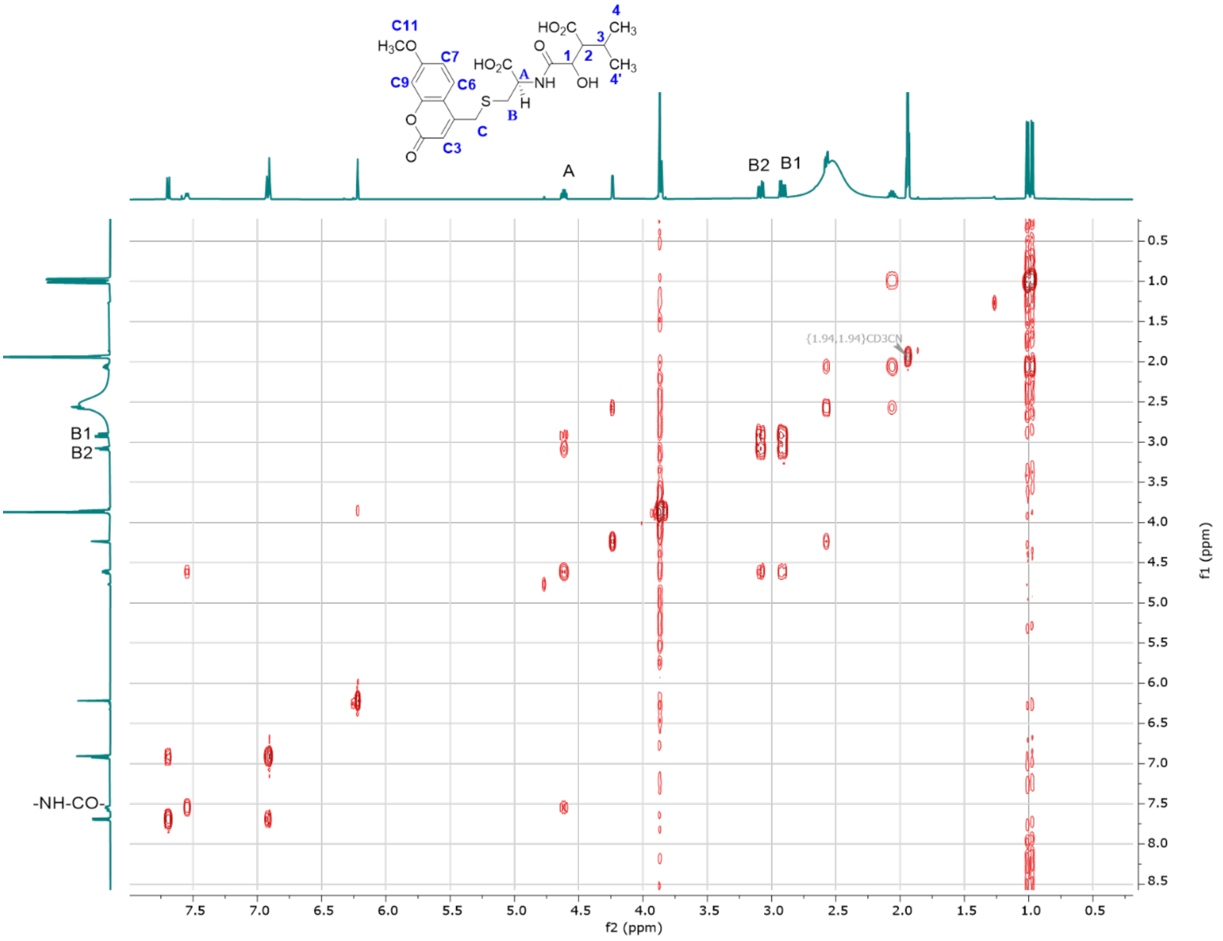

Figure S8. COSY NMR of Compound **4a**. The signal assigned to the proton B on amide bond (δ 7.55 ppm) can see the alpha proton A on cysteine (δ 4.61 ppm), suggesting that the proton B and proton A are attached to two neighbouring carbons

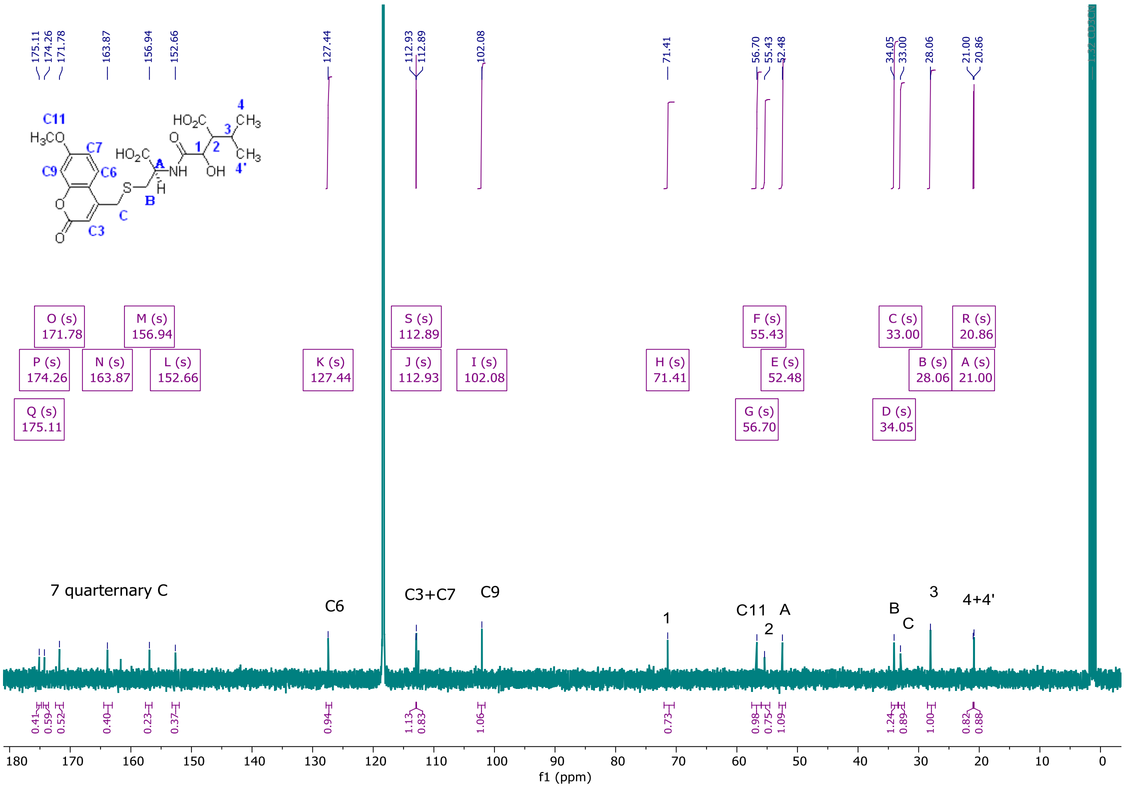

Figure S9. ^{13}C NMR of Compound **4a** (126 MHz, CD_3CN) δ 175.11, 174.26, 171.78, 163.87, 156.94, 152.66, 127.44, 112.93, 112.89, 102.08, 71.41, 56.70, 55.43, 52.48, 34.05, 33.00, 28.06, 21.00, 20.86.

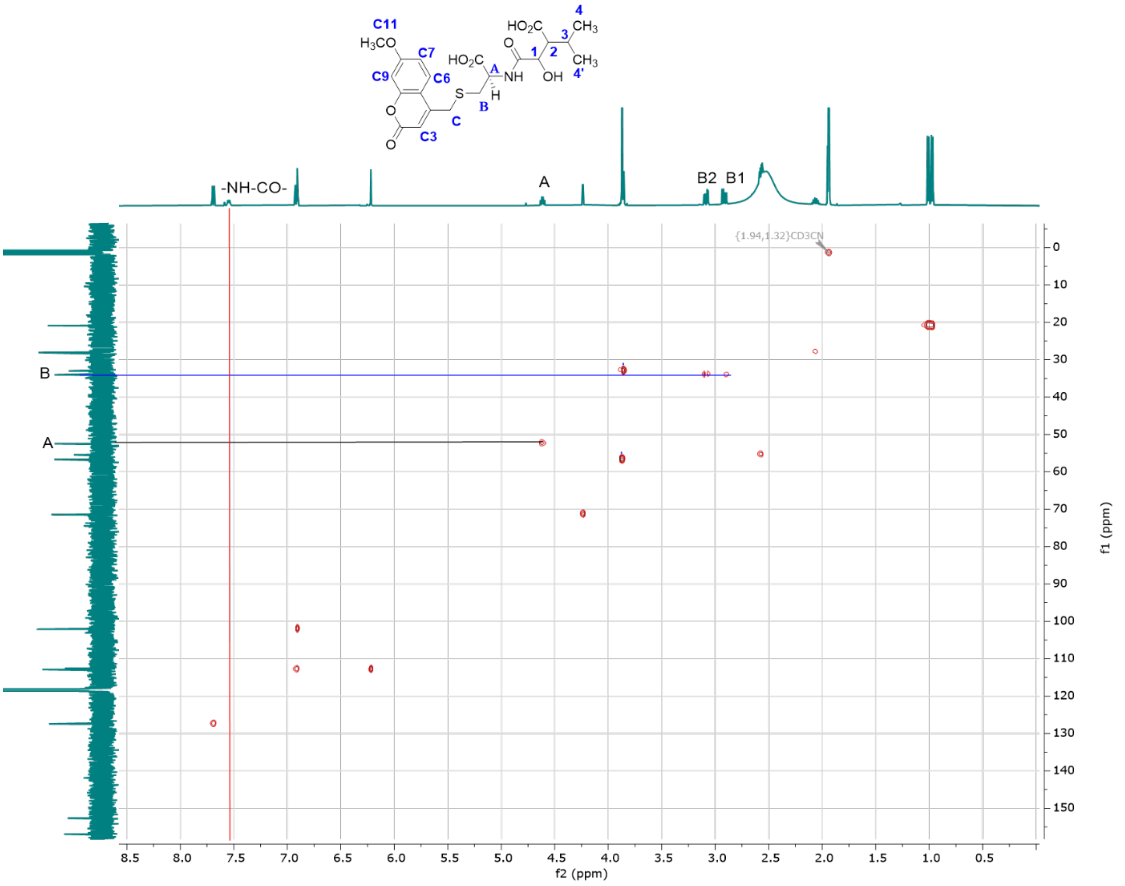

Figure S10. HMQC NMR of Compound **4a**. This spectrum shows the correlation between the carbon and the attached protons of the proposed structure. Proton B1 and B2 both connect to carbon B (blue line). The proton on the designated amide bond does not correlate to any carbon directly (red line).

Isopropyl Malic Acid and Coenzyme A

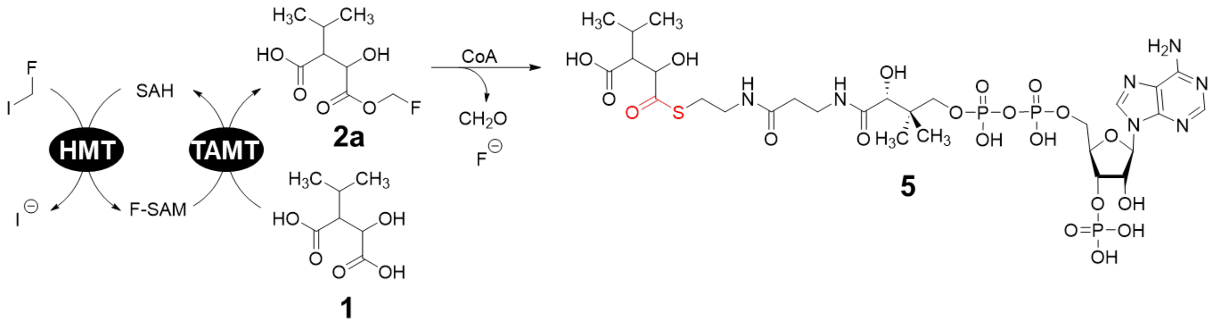

Scheme S1.

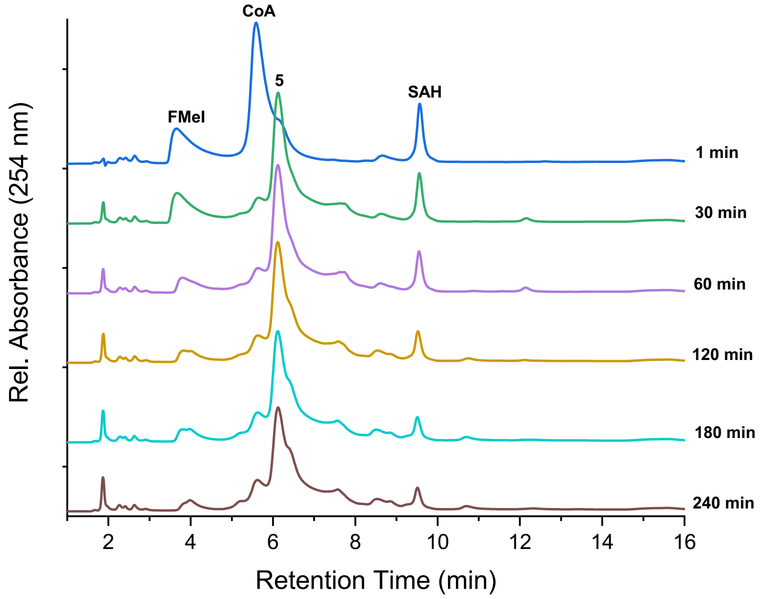

Figure S11. HPLC Chromatogram of Reaction Progress between FMeI, **1** and coenzyme A (CoA). **2a** reacted with CoA. CoA was completely converted to a new product after 30 min, and the product (**5**) was stable. 2 mM of isopropyl malic acid, 10 mM FMeI and 2 mM coenzyme A sodium salt (CoA) was incubated with 2 mM TCEP, 50 µM of HMT, 50 µM of TAMT, 50 µM of SAH at 25 °C in 50 mM sodium phosphate buffer, pH 8.0. The reaction is quenched at 1, 15, 30, 60, 120, 180, 240 min by mixing 20 µL of reaction with 20 µL of 1 M H_3PO_4. The mixture was centrifuged at 14000 × g. 10 µL of the supernatant was analyzed by HPLC using a cation exchange HPLC column 150 × 4.6 mm (Luna 5 µm SCX 100 Å). The mobile phase was constituted of buffer A (20 mM H_3PO_4 in deionized water) and buffer B (20 mM H_3PO_4, 1 M NaCl in deionized water, filtered through 0.22 µm MF-Millipore MCE membrane). HPLC buffer gradient: 0-2 min, 2% B; 12 min, 70% B; 13-16 min, 99% B; 17-19 min, 2% B.

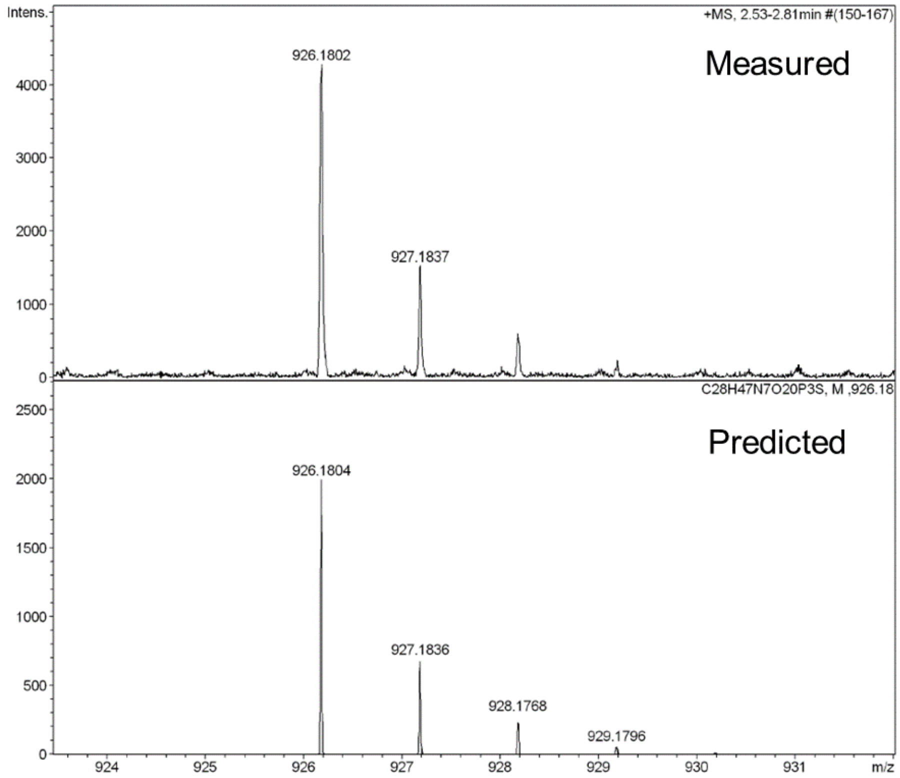

Figure S12. HR-ESI-MS spectrum of IPMA-CoA adduct. The predicted m/z of compound **5** $[M+H]^+$ ($C_{28}H_{47}N_7O_{20}P_3S^+$) is 926.1804, the measured m/z is 926.1802.

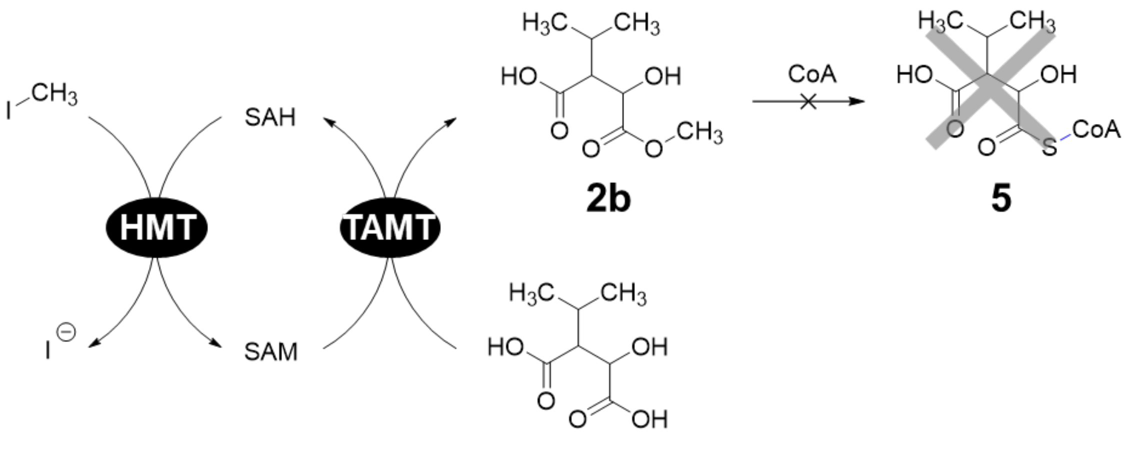

Scheme S2.

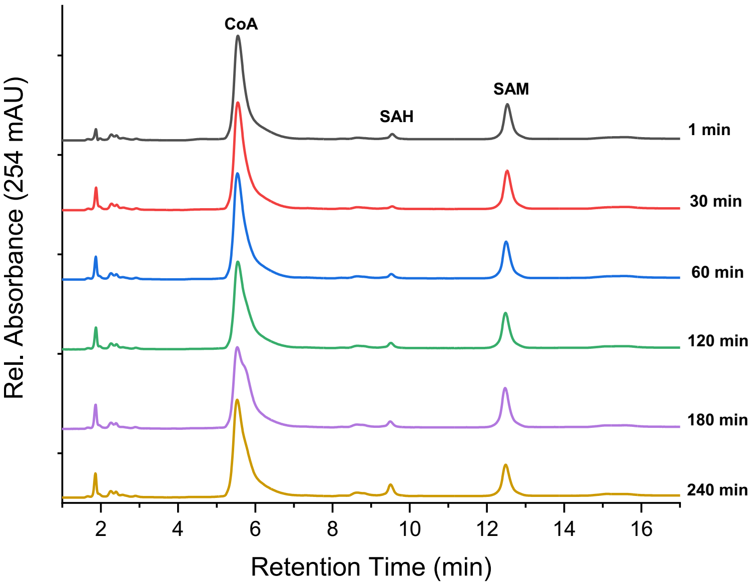

Figure S13. HPLC analysis of the reaction between MeI, **1** and CoA shows that **2b** does not react with CoA. CoA was not derivatized in the observed time period. 2 mM of isopropyl malic acid, 10 mM MeI and 2 mM coenzyme A sodium salt (CoA) was incubated with 2 mM TCEP, 50 µM of HMT, 50 µM of TAMT, 50 µM of SAH at 25 °C in 50 mM sodium phosphate buffer, pH 8.0. The reaction is quenched at 1, 15, 30, 60, 120, 180, 240 min by mixing 20 µL of reaction with 20 µL of 1 M H_3PO_4. The preparation of the HPLC sample and the HPLC conditions are identical to Figure S12.

Indole-3-acetic acid conjugation with cysteine

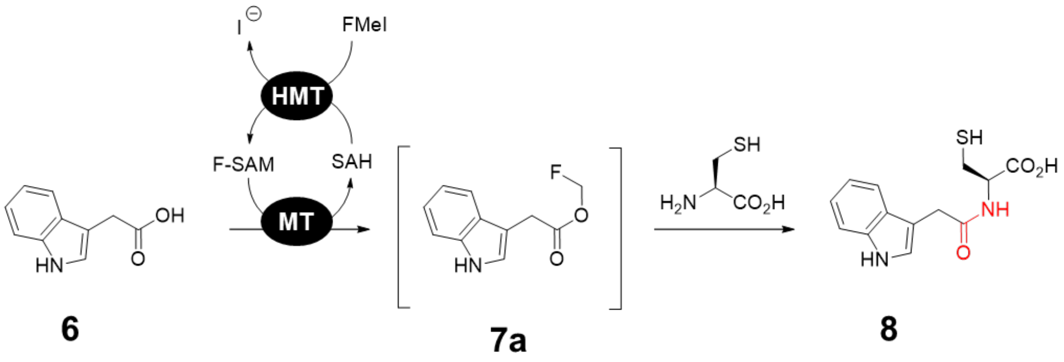

Scheme S3.

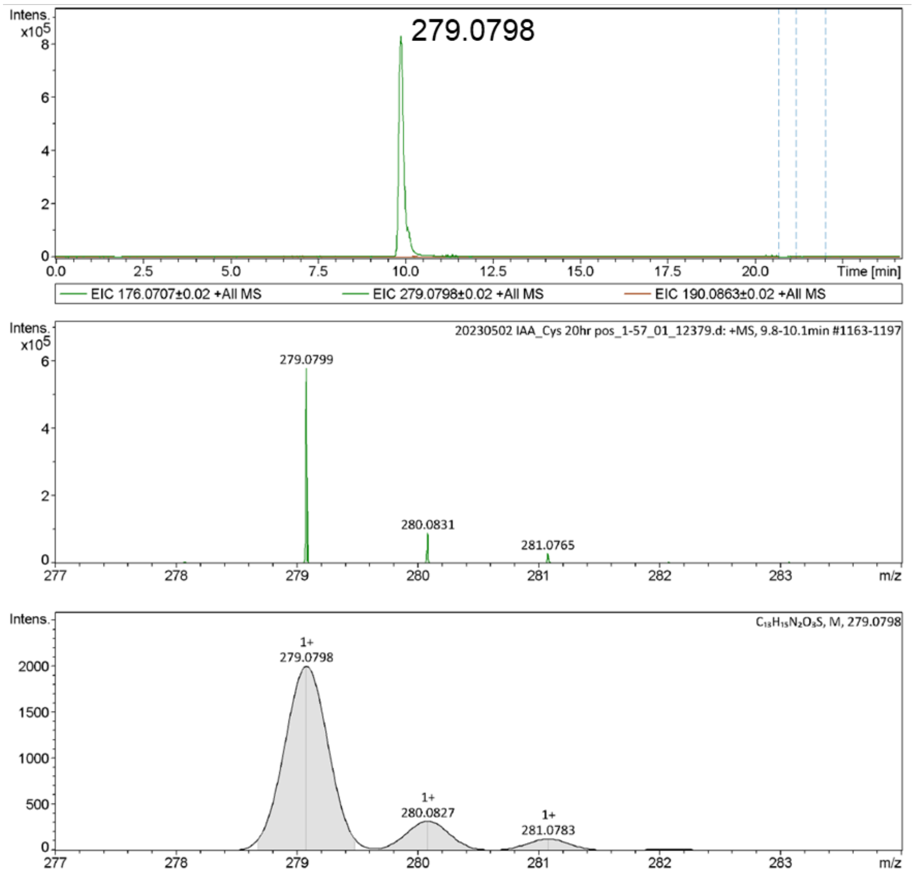

Figure S14. HR-ESI-MS spectrum confirming the formation of compound **8**. **Top:** EIC chromatogram of compound **8** (279.0798 ± 0.02) extracted from the measurement of the reaction mixture after 20 hours. Compound **6** could not be detected (176.0707 ± 0.02). **Middle:** averaged spectrum from the EIC chromatogram, measured m/z is 279.0799. Bottom: calculated m/z pattern of compound **8** is 279.0798, based on the protonated form $[C_{13}H_{15}O_3N_2S]^+$.

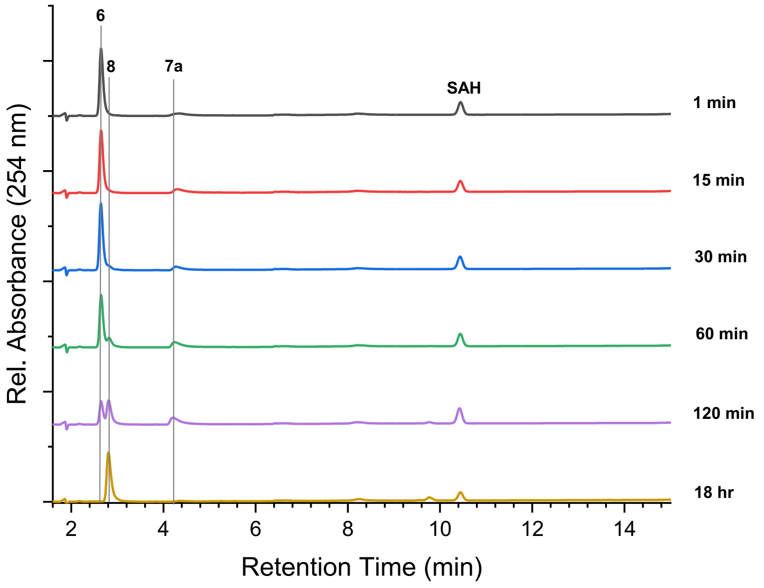

Figure S15. HPLC chromatogram monitoring the ligation between FMeI, cysteine and **6**. Compound **6** was converted completely to **8** via the formation of **7a.** 1 mM of **6** was incubated with 4 mM FMeI, 40 µM SAH, 20 µM HMT, 20 µM IAAMT, 4 mM cysteine, 5 mM TCEP, 1 mM $MgCl_2$ in 200 µL of Na phosphate buffer (pH 7.8). Compound **7a** did not accumulate. A new species eluted after **6**. This species was identified as **8** (Figure 14). After 1, 15, 30, 60, 120 min and 18 hr, 5 µL of the reaction was quenched by mixing with 5 µL of 1 M H_3PO_4. This mixture was analysed by cation exchange HPLC with UV. The mobile phase was constituted of buffer A (20 mM H_3PO_4 in deionized water) and buffer B (20 mM H_3PO_4, 1 M NaCl in deionized water, filtered through 0.22 µm MF-Millipore MCE membrane). HPLC buffer gradient: 0-2 min, 2% B; 12 min, 60% B; 15-18 min, 99% B; 19-22 min, 2% B.

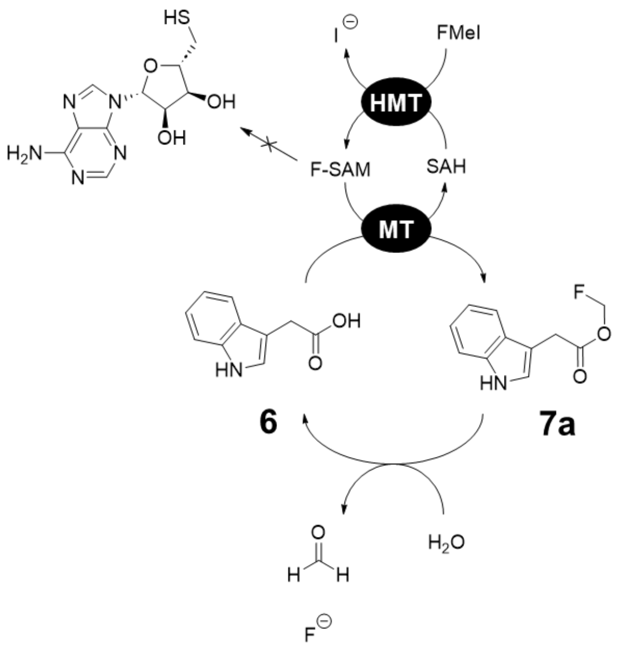

Scheme S4.

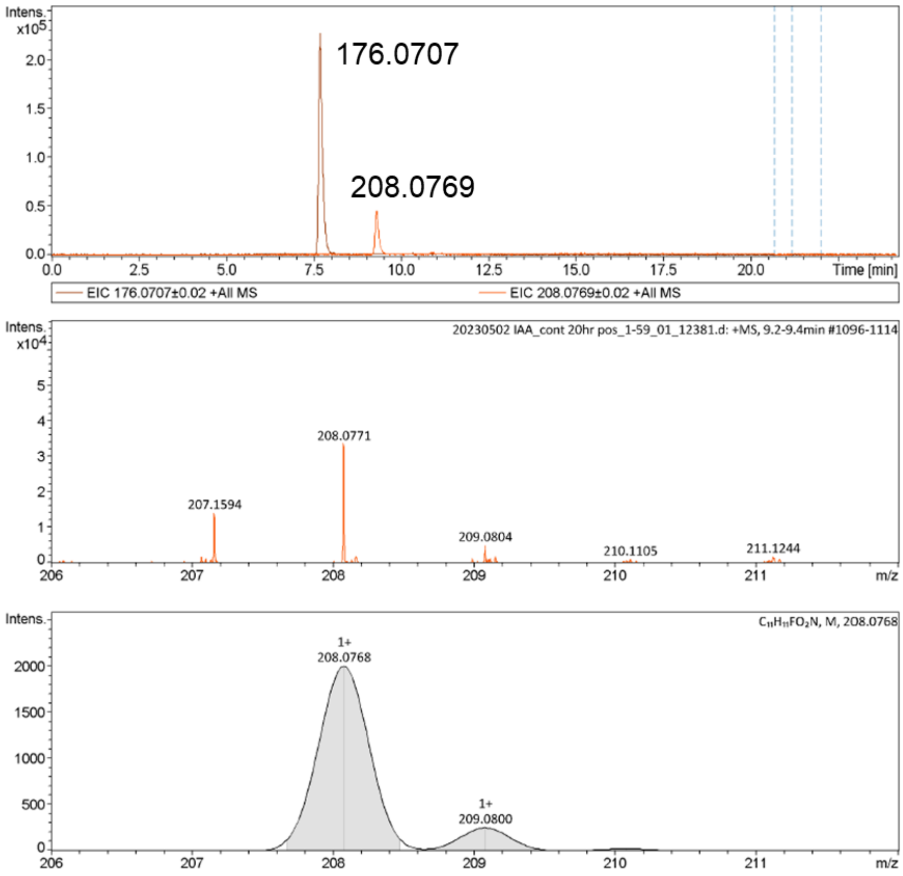

Figure S16. HR-ESI-MS spectrum confirming the transient formation of **7a**. **Top:** EIC chromatogram of compound **6** (176.070 ± 0.02) and compound **7a** (208.0769 ± 0.02) extracted from the measurement of the reaction mixture after 20 hours. **Middle:** averaged spectrum from the EIC chromatogram, measured m/z is 208.0771. **Bottom:** calculated m/z pattern of compound **8** (208.0768), based on the protonated form $[C_{11}H_{11}FO_2N]^+$.

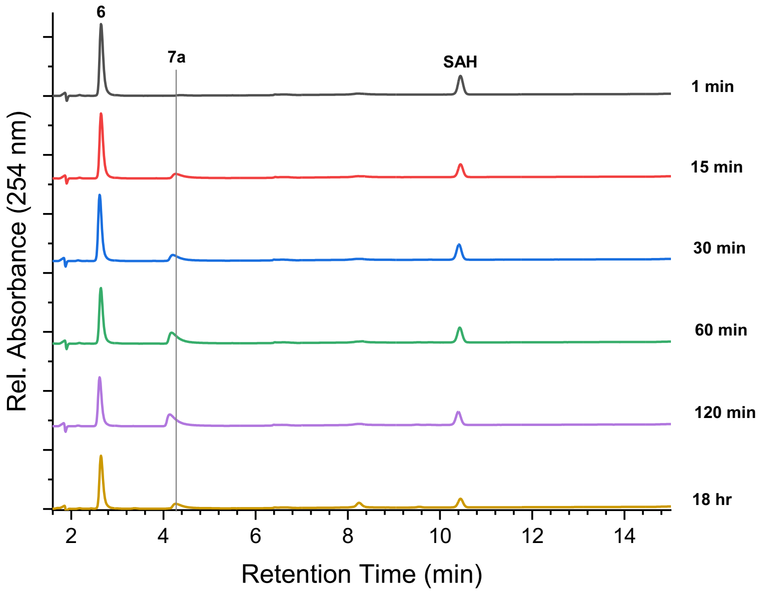

Figure S17. HPLC chromatogram monitoring the reaction between FMeI and **6** in the absence of Cys. 1 mM of **6** was incubated with 4 mM FMeI, 40 µM of SAH, 20 µM of HMT, 20 µM of IAAMT, 1 mM $MgCl_2$, 5 mM TCEP in 50 mM Na phosphate buffer pH 8.0 at 25 °C. The work-up and analytical procedure is identical to Figure S15. These observations show that **6** was transformed to **7a**, but that this F-methyl ester did not accumulate.

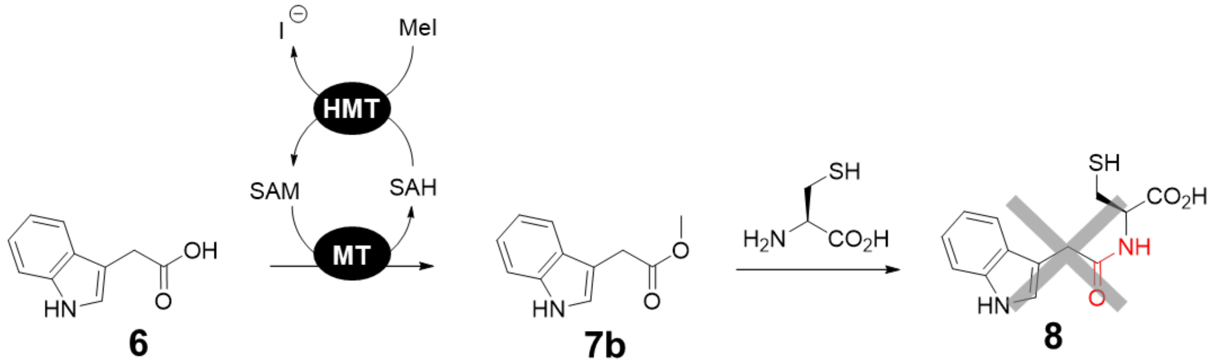

Scheme S5.

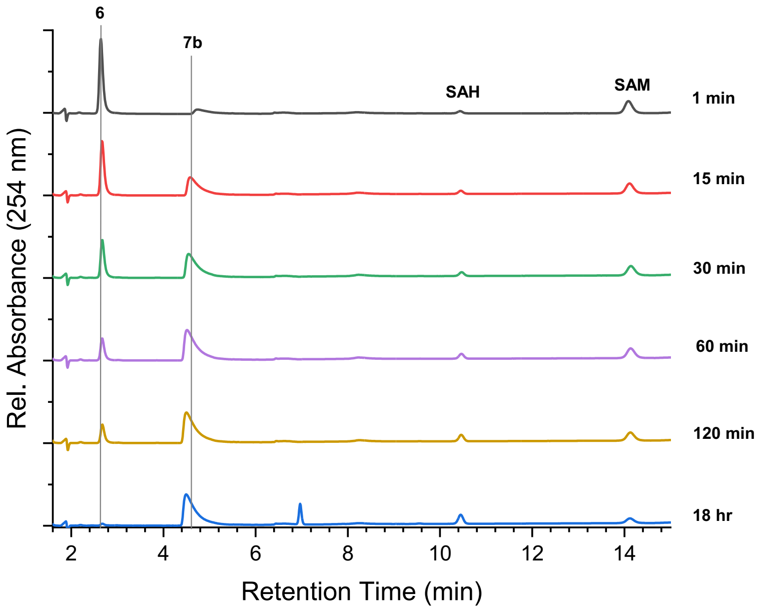

Figure S18. HPLC chromatogram monitoring the reaction between MeI and **6**. 1 mM of IAA was incubated with 4 mM cysteine, 5 mM TCEP, 4 mM MeI, 40 µM SAH, 20 µM HMT, 20 µM IAAMT, 1 mM $MgCl_2$ in 200 µL of Na phosphate buffer (pH 7.8). Compound **6** was completely transformed to **7b** after 18 hours. No conjugate **8** could be detected. The work-up and analytical procedure is identical to Figure S15.

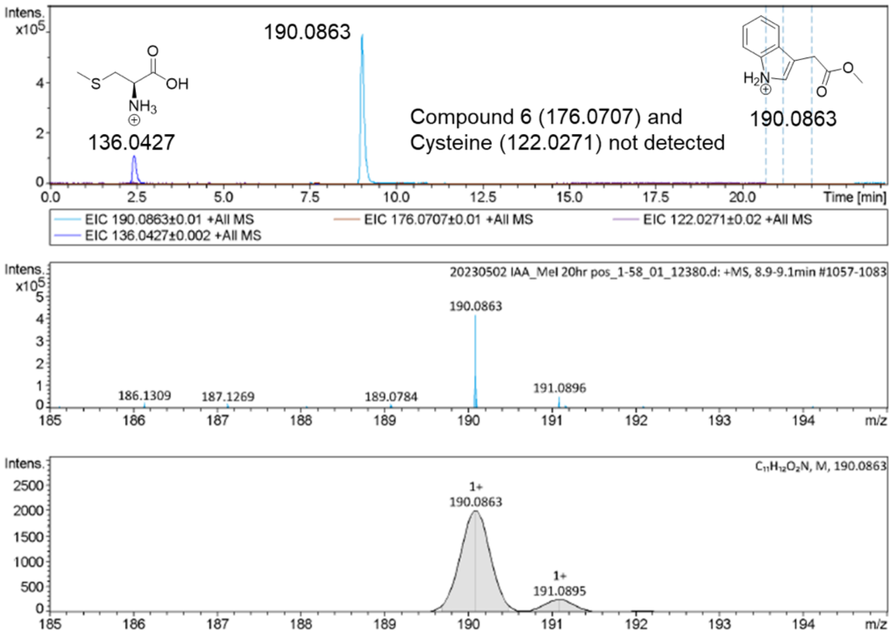

Figure S19. HR-ESI-MS spectrum confirming the formation of **7b**. **Top:** EIC chromatogram of compound **7b** (190.0863 ± 0.01) extracted from the measurement of the reaction mixture after 20 hours. Cysteine (122.0271 ± 0.01) was not detected. Methylated cysteine (136.0427 ± 0.01) was found. **Middle:** averaged spectrum from the EIC chromatogram, measured m/z is 190.0863. **Bottom:** calculated m/z pattern of compound **7b** is 190.0863.

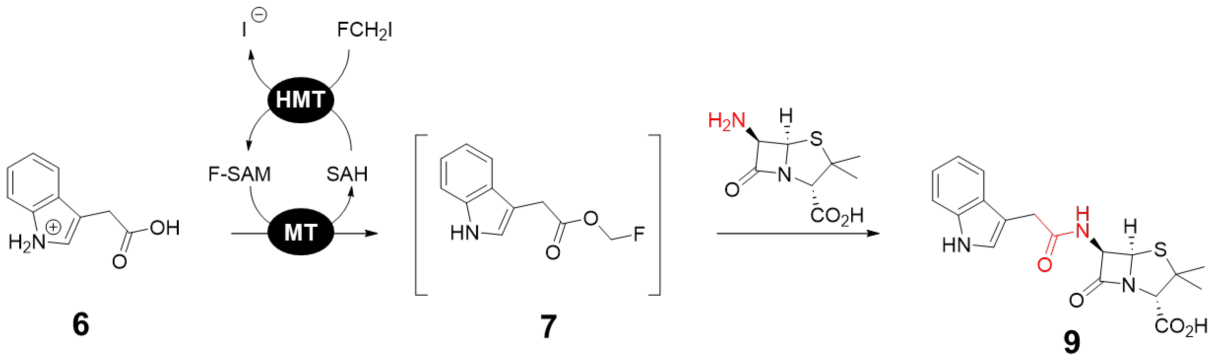

Scheme S6.

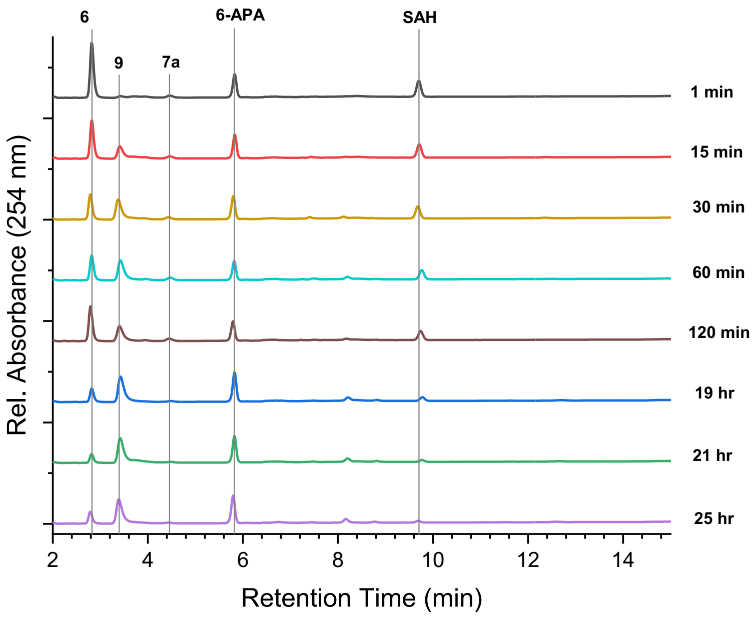

Figure S20. HPLC chromatogram monitoring the reaction between compound **6** and 6-APA. A 200 µL solution of 50 mM sodium phosphate buffer at pH 8, 25 °C, 1 mM of **6** was incubated with 10 mM of 6-aminopenicillanic acid (6-APA), 4 mM FMeI, 20 µM HMT, 20 µM TAMT, 40 µM SAH, 2 mM $MgCl_2$. 2 mM of FMeI was added after 19 hr of incubation. At 1, 15, 30, 60, 120 min, 19 and 25 hr, 5 µL of the reaction mixture was quenched. The work-up and analytical procedure is identical to Figure S14.

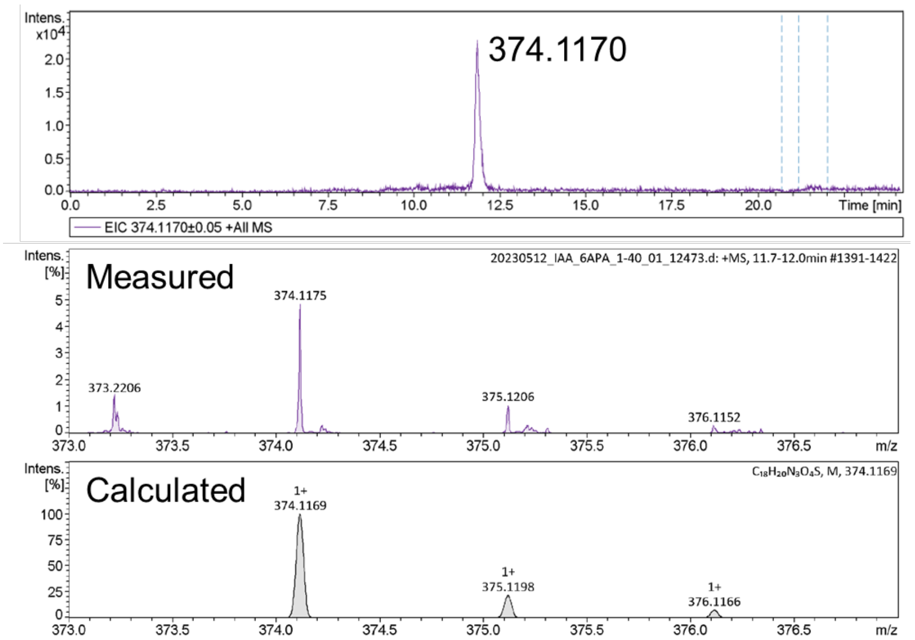

Figure S21. Top: EIC chromatogram of compounds **9** (374.1170 ± 0.05) extracted from the measurement of the reaction mixture after 25 hrs. **Middle:** found m/z of $[C_{18}H_{20}N_3O_4S]^+$ is 374.1175. **Bottom:** calculated m/z pattern of compound **9** is 374.1169.

Functionalization of isoAsp-containing peptides with Methoxyamine

To a 50 uL reaction containing 20 uM isoAsp peptide, 50 µM SAH, 50 µM HMT, 2.75 µM PIMT and 5 mM FMeI or MeI in 0.1 M sodium phosphate buffer (pH 7.0) was added 50 uL of 2 M methoxyamine at pH 7 and incubated at room temperature. At indicated times, 5 uL of the reaction were quenched with 45 uL 50% MeCN, 1% formic acid in water and 1 uL thereof were analysed by RP-UPLC-MS (Ultra Performance Liquid Chromatography Mass Spectrometry) on a Xevo G2-XS QTof MS (Quadrupole Time of Flight Mass Spectrometer).

Relative amounts of the species were estimated by the normalised peak intensities and fit to a two-step sequential reaction. To facilitate comparison between the two reactions, the conversion of the isoAsp-peptide to the succinimide form was treated as a single step. In the case of F-methylation (top), the condensation of the F-methyl ester is much faster and the rate is dominated by the initial enzyme-catalysed F-methylation, defined as k_1. Conversely, PIMT-dependent methylation is fast (bottom), but the resulting methyl ester only slowly condenses into a succinimide intermediate with a rate of k_1'. The nucleophilic ring opening step is described by k_2 in both reaction sequences.

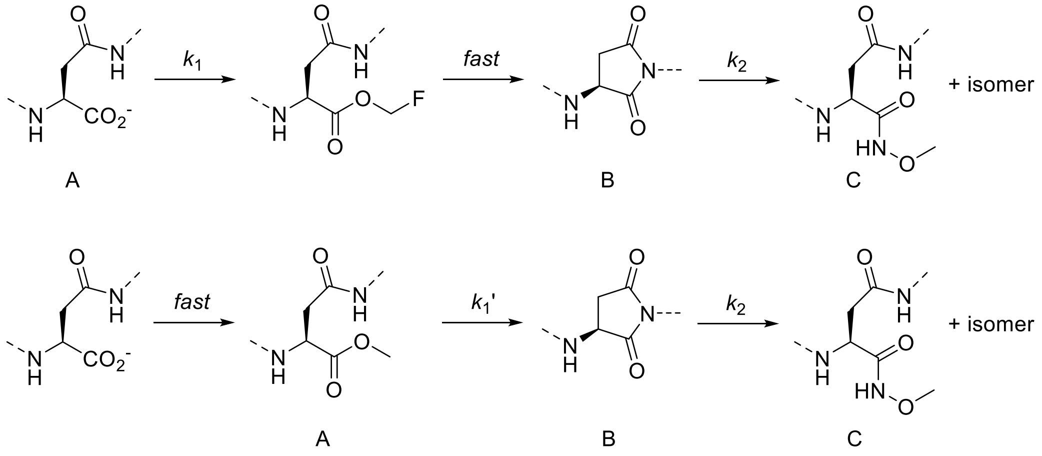

Scheme S7.

The reaction progress was fit to the following set of equations where A(t), A_0 and A_f are the relative concentrations of the isoAsp peptide (F-methylation) or the methyl ester (methylation) as a function of time, at the beginning and at the end of the reaction, respectively. B(t) and C(t) correspond to the relative concentrations of the succinimide and methoxyamine adduct, respectively (Figure S22).

$$A(t) = (A_0 - A_f)e^{-k_1 t} + A_f,$$

$$B(t) = (A_0 - A_f)\frac{k_1}{k_2 - k_1}(e^{-k_1 t} - e^{-k_2 t}), \text{ and}$$

$$C(t) = (A_0 - A_f)(1 + \frac{k_1 e^{-k_2 t} - k_2 e^{-k_1 t}}{k_2 - k_1}),$$

Functionalization of isoAsp-containing peptides with Biotin oxyamine: A F-methylation reaction containing 20 µM isoAsp peptide (**10**), 50 µM SAH, 50 µM HMT, 2.75 µM PIMT and 5 mM FMeI was prepared in 50 uL 0.1 M sodium phosphate buffer (pH 7.0). 10 uL of this reaction were immediately added to 5 uL approximately 0.3 M biotin-PEG$_3$-oxyamine (Sigma QBD11100) at pH 7. At indicated times, 5 uL of the reaction were quenched with 45 uL 50% MeCN, 1% formic acid in water and 1 uL thereof were analysed by RP-UPLC-MS on a Xevo G2-XS QTof MS.

<u>IsoAsp Peptide synthesis</u>: A peptide containing an isoAsp residue (derived from residues 20-39 of human p53: Ac-SDLWKLLPE<u>X</u>NVLSPLPSQA-NH2, where <u>X</u> is isoAsp), was synthesized on a rink amide resin using microwave assisted Fmoc-SPPS on a Biotage Initiator+ Alstra as described previously.[4] Couplings were performed at 70 °C using DIC/K-Oxyma. Fmoc-deprotections were performed at room temperature with 20% piperidine in DMF. After the synthesis, the N-terminus was acetylated with acetic anhydride and DIPEA. The peptide was subsequently cleaved from the resin by treatment with 95% TFA, 2.5% triisopropylsilane (TIS), 2.5% H_2O. Peptides were precipitated with cold diethyl ether, dissolved in 50% B and lyophilized. The peptide was dissolved in 20% MeCN and purified via RP-HPLC on a preparative C18 column with a gradient of 25-45% MeCN containing 0.1% TFA in water containing 0.1% TFA (Expected Mass: 2262.190 Da, Found: 2262.183 Da).

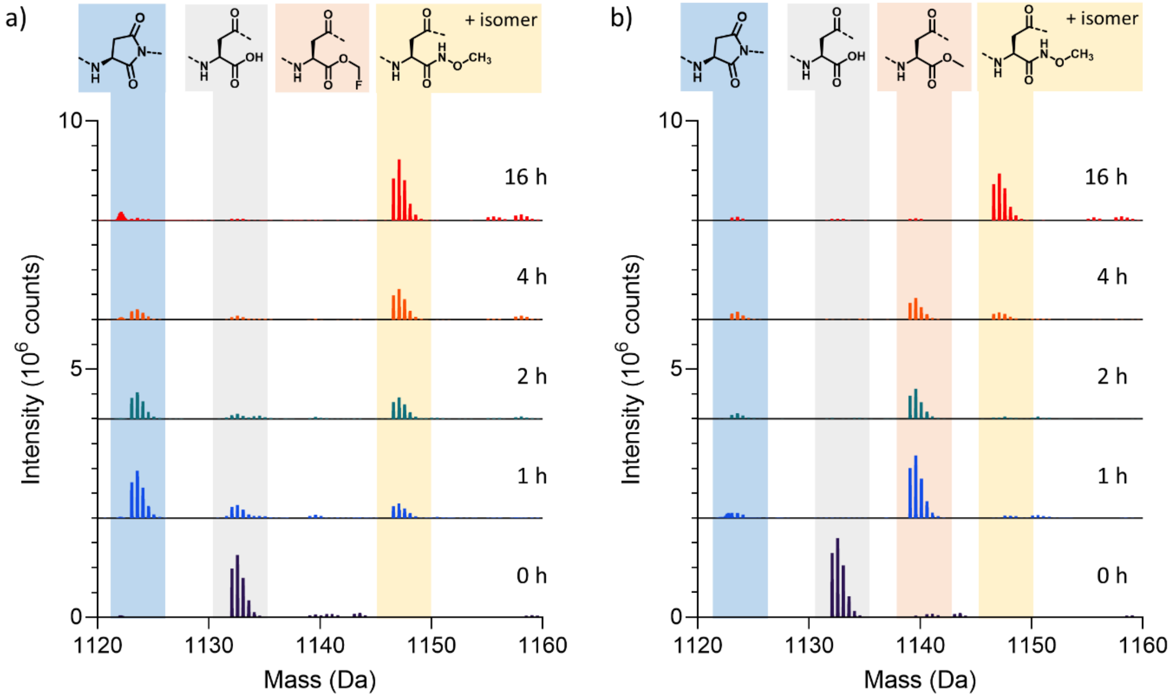

Figure S22. ESI-ToF MS spectra of isoAsp-derived peptides upon chemoenzymatic functionalisation with a F-methylation (a) or methylation (b) cascade. Observed species include the starting material (gray, $[C_{103}H_{165}N_{25}O_{32}]^{2+}$ calcd: m/z 1132.604, found: 1132.595), methyl ester (salmon, $[C_{104}H_{167}N_{25}O_{32}]^{2+}$ calcd: m/z 1139.612, found: 1139.604), succinimide (blue, $[C_{103}H_{163}N_{25}O_{31}]^{2+}$ calcd: m/z 1123.598, found: 1123.590) and methoxyamine adduct (yellow, $[C_{104}H_{168}N_{26}O_{32}]^{2+}$ calcd: m/z 1147.117, found: 1147.106). The F-methyl ester ($[C_{104}H_{166}FN_{25}O_{32}]^{2+}$ calcd: m/z 1148.607) was not observed.

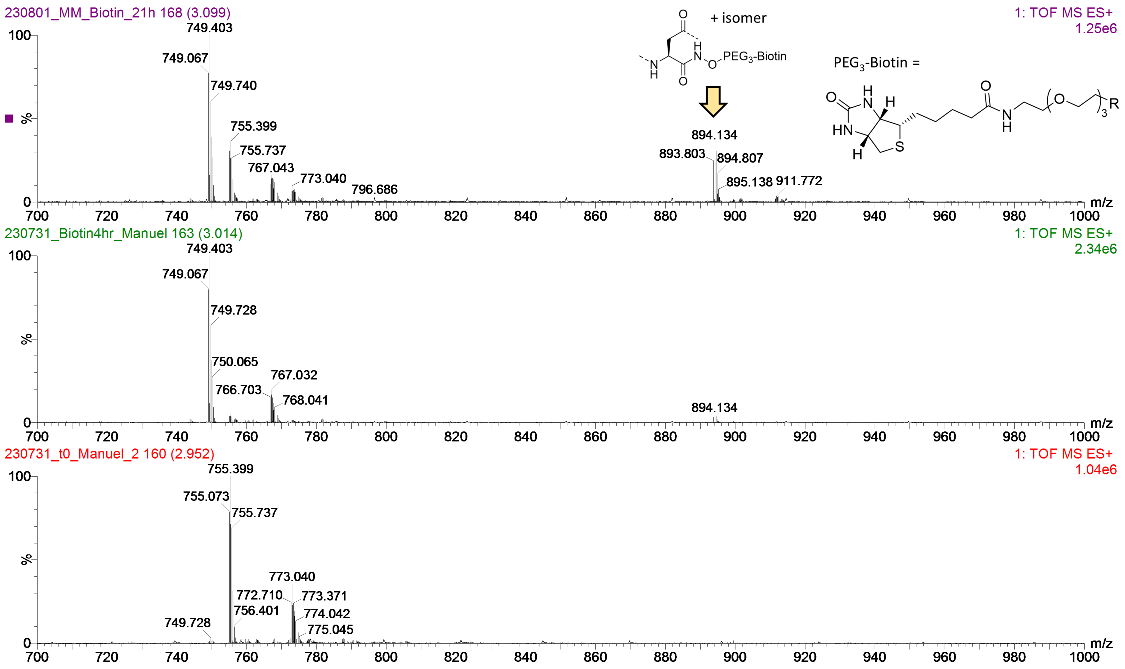

Figure S23. ESI-ToF MS spectra of the isoAsp-peptide biotinylation reactions. A time course is shown from 0h (bottom), 4h (middle) to 21h (top). Observed species include the starting material ($[C_{103}H_{166}N_{25}O_{32}]^{3+}$ calcd: m/z 755.405, found: 755.399) and its NH_4Cl adduct ($[C_{103}H_{170}ClN_{26}O_{32}]^{3+}$ calcd: m/z 773.073, found: 773.040), succinimide ($[C_{103}H_{164}N_{25}O_{31}]^{3+}$ calcd: m/z 749.401, found: 749.403) and biotin-PEG3-methoxyamine adduct ($[C_{121}H_{198}N_{29}O_{37}S]^{3+}$ calcd: m/z 894.141, found: 894.134).

The conjugation between GFP and cysteine

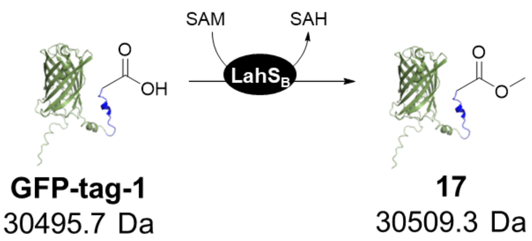

Scheme S8.

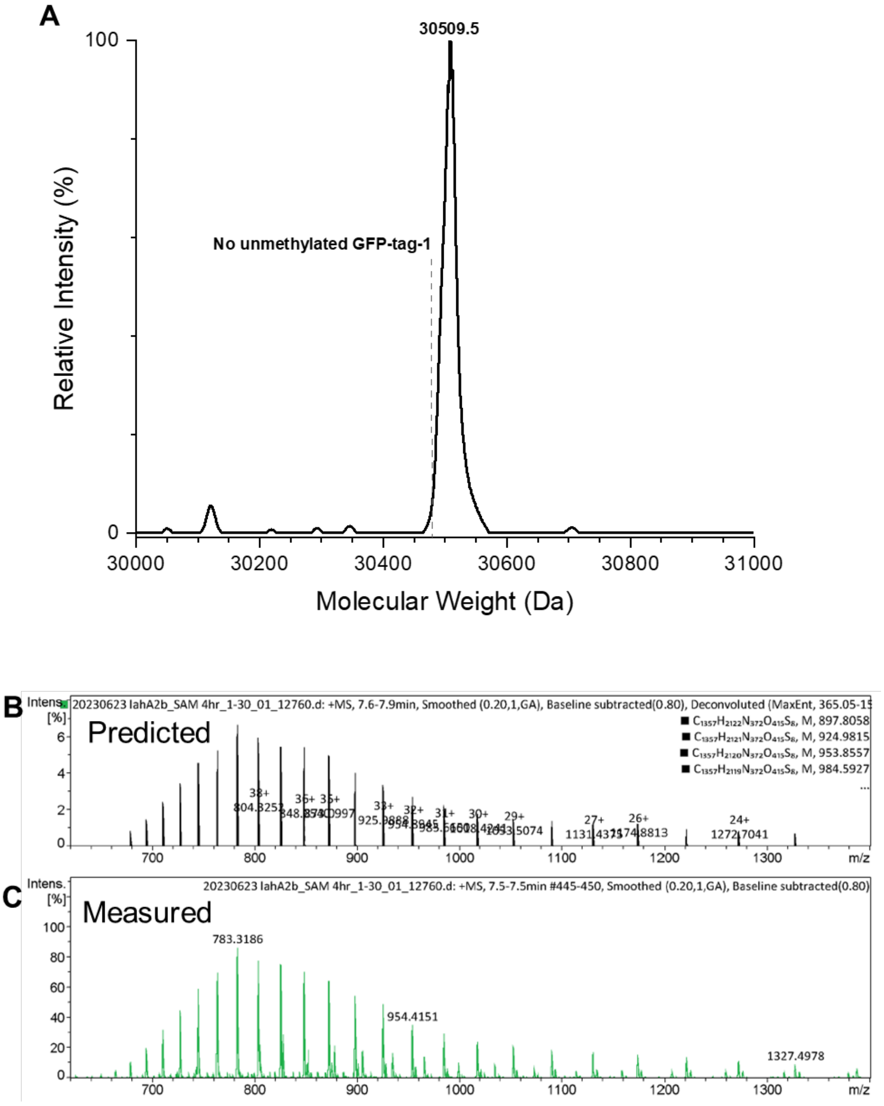

Figure S24. HR-ESI-MS spectrum of methylated GFP-tag-1. **A:** deconvoluted mass spectrum of a 4-hr methylation reaction. Unmethylated GFP-tag-1 was not detectable (dashed line). **B:** predicted m/z spectrum of methylated GFP-tag-1 (**17**). **C**: measured m/z spectrum. The sample for this measurement was prepared as follows: A 200 µL solution of 50 mM sodium phosphate buffer at pH 8.0, containing 20 µM GFP-tag-1, 1 mM *S*-adenosyl methionine (SAM) and 10 µM LahS$_B$ was incubated at 25 °C. After 4 hrs, 10 µL of the reaction was quenched with 5 µL of 10 % formic acid. These solutions were submitted for analysis by HR-ESI-MS.

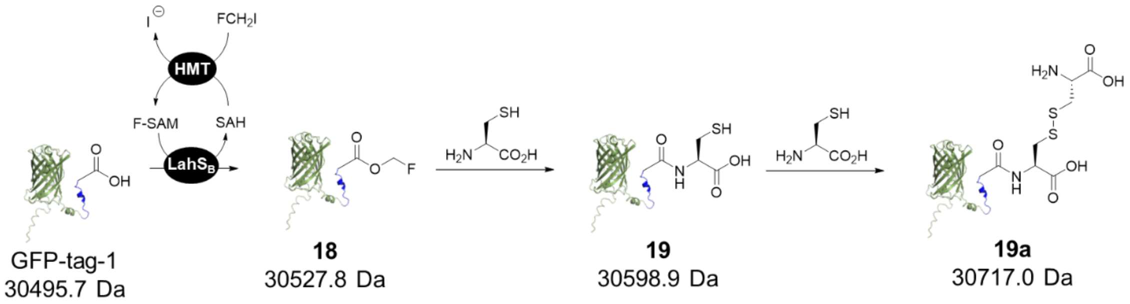

Scheme S9.

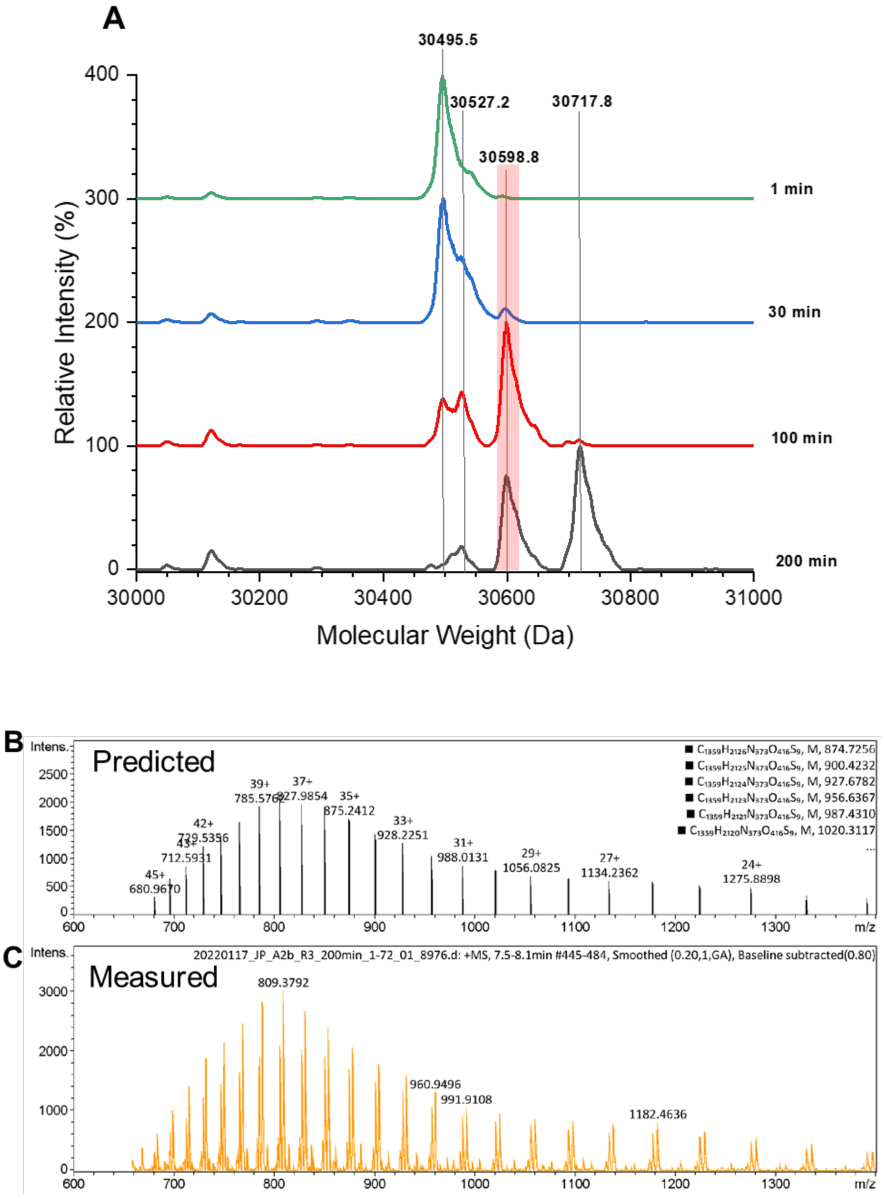

Figure S25. HR-ESI-MS spectra showing the ligation between GFP-tag-1 and cysteine. **A:** deconvoluted mass spectra of the ligation reaction after 1, 30, 100 and 200 min. **B:** predicted spectrum of ligation product **19**. **C:** measured spectrum of the reaction after 200 min. The samples for this measurement were prepared as follows: A 200 µL solution of 50 mM sodium phosphate buffer at pH 8.0, containing 20 µM of GFP-tag-1, 2 mM cysteine, 2 mM TCEP.HCl, 1 mM FMeI, 20 µM SAH, 10 µM HMT and 10 µM $LahS_B$ was incubated at 25°C. After 1 min, 30 min, 100 min and 200 min, 10 µL of the reaction was quenched with 5 µL of 10 % formic acid. These solutions were submitted for analysis by HR-ESI-MS.

Optimizing parameter: pH

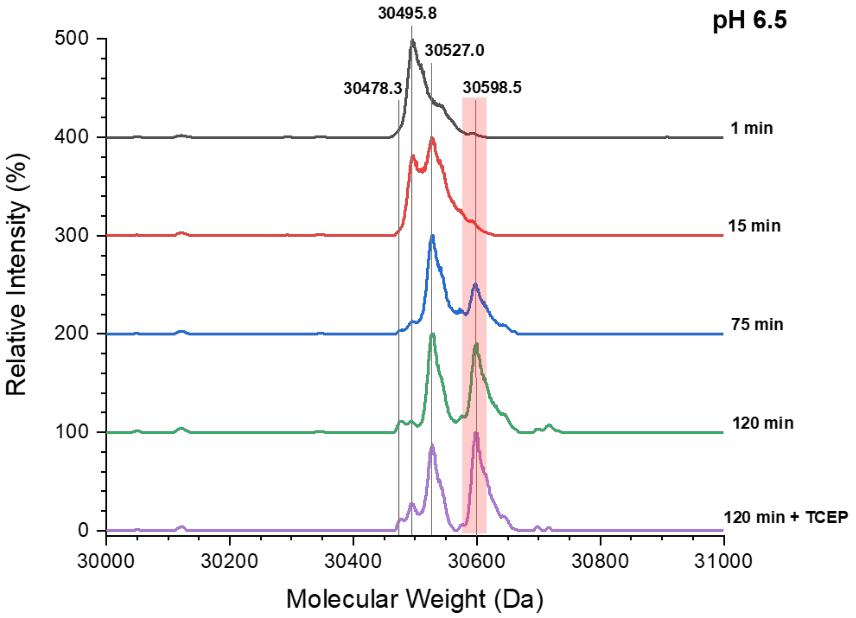

Figure S26. HR-ESI-MS spectra showing the ligation between GFP-tag-1 and cysteine at pH 6.5. Deconvoluted mass spectra of the ligation reaction after 1, 15, 75, 120 min and 120 min with TCEP. The samples for this measurement were prepared as follows: A 200 µL solution of 50 mM sodium phosphate buffer at pH 6.5, containing 40 µM of GFP-tag-1, 10 µM of HMT, 10 µM of $LahS_B$, 20 µM of SAH, 1 mM of FMeI, 2 mM of cysteine, and 2 mM TCEP was incubated at 25°C. After 1, 15, 75 and 120 min, 5 µL reaction aliquot was quenched with 10% formic acid and submitted to analysis by HR-ESI-MS. The 120 min sample was supplemented with an additional dose of 2 mM TCEP and submitted again for analysis by HR-ESI-MS.

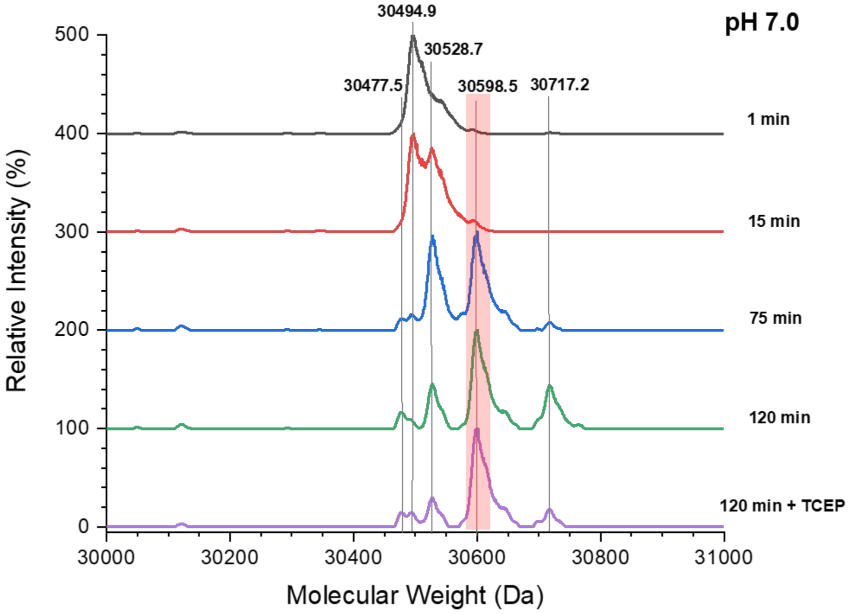

Figure S27. HR-ESI-MS spectra showing the ligation between GFP-tag-1 and cysteine at pH 7.0. The reaction conditions are as described in Figure S26, except the buffer pH is 7.0.

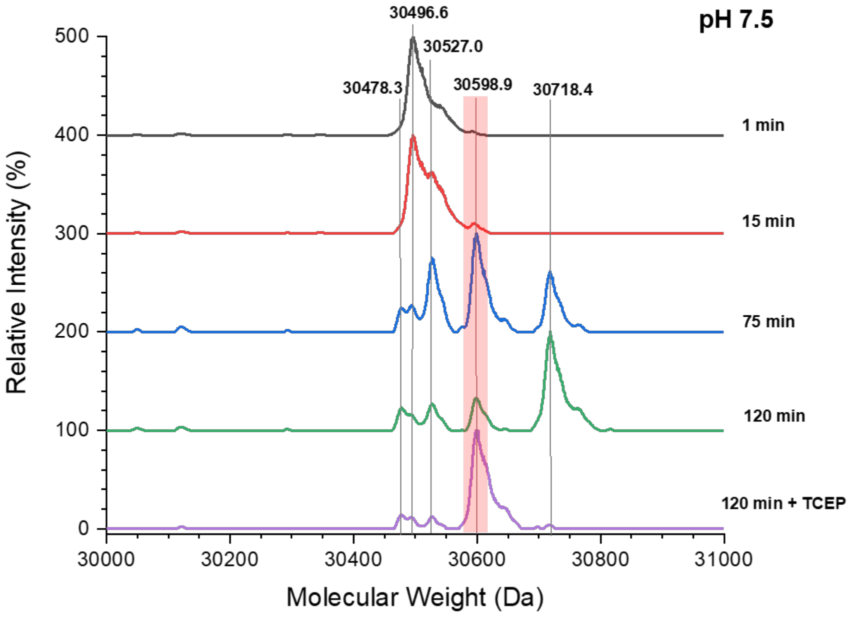

Figure S28. HR-ESI-MS spectra showing the ligation between GFP-tag-1 and cysteine at pH 7.5. The reaction conditions are as described in Figure S26, except the buffer pH is 7.5.

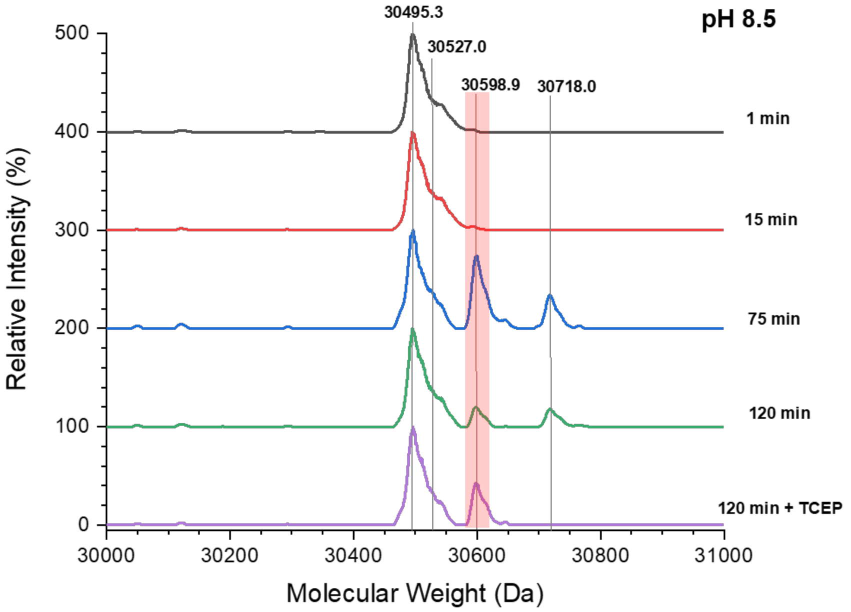

Figure S29. HR-ESI-MS spectra showing the ligation between GFP-tag-1 and cysteine at pH 7.5. The reaction conditions are as described in Figure S26, except the buffer pH is 8.5.

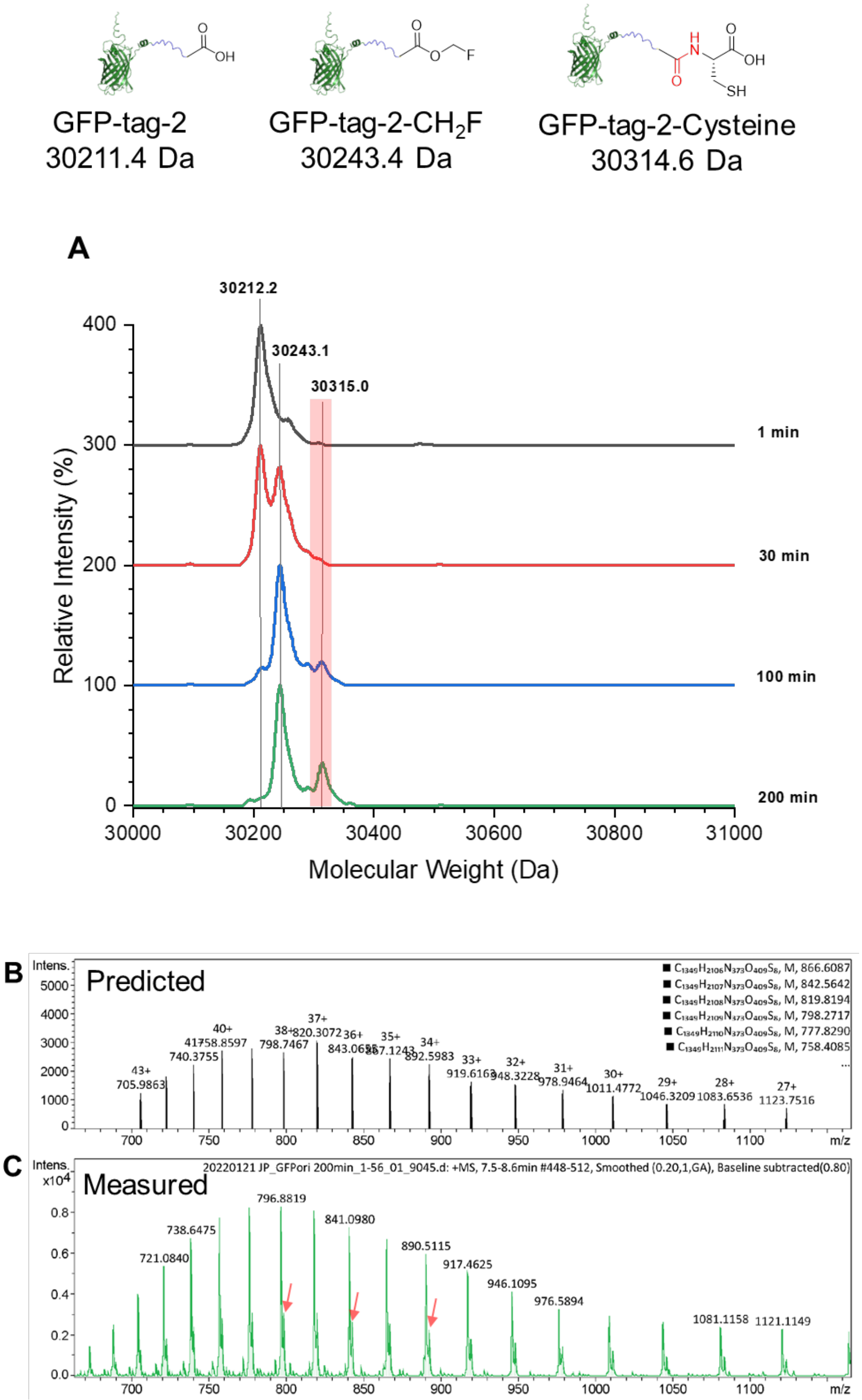

Figure S30. HR-ESI-MS spectra showing the ligation between GFP-tag-2 and cysteine. **A:** deconvoluted mass spectra of the ligation reaction after 1, 30, 100 and 200 min. **B:** predicted spectrum of ligation product GFP-tag-2-Cysteine. **C:** measured spectrum of the reaction after 200 min. The samples for this measurement were prepared as follows: A 200 µL solution of 50 mM sodium phosphate buffer at pH 8.0, containing 20 µM of GFP-tag-2, 2 mM cysteine, 2 mM TCEP.HCl, 1 mM FMeI, 20 µM SAH, 10 µM HMT and 10 µM $LahS_B$ was incubated at 25°C. After 1 min, 30 min, 100 min and 200 min, 10 µL aliquots of the reaction were quenched with 5 µL of 10 % formic acid. These solutions were submitted for analysis by HR-ESI-MS. These results show that $LahS_B$ F-methylated GFP-tag-2 efficiently, but that less than 30 % of F-methylated GFP-tag-2 ligated to cysteine.

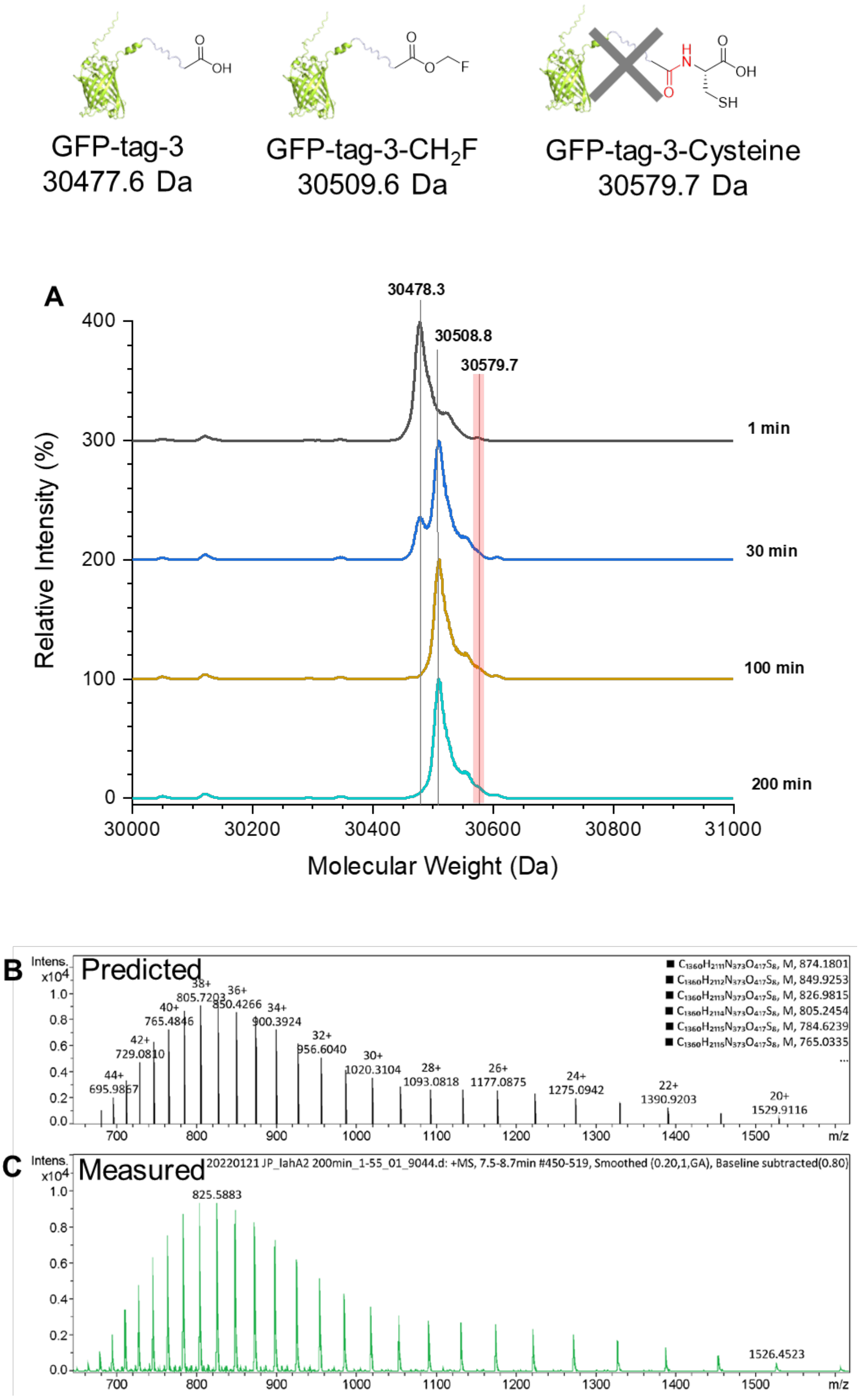

Figure S31. HR-ESI-MS spectra showing the ligation between GFP-tag-3 and cysteine. **A:** deconvoluted mass spectra of the ligation reaction after 1, 30, 100 and 200 min. **B:** predicted spectrum of ligation product GFP-tag-3-Cysteine. **C:** measured spectrum of the reaction after 200 min. The samples for this measurement were prepared as follows: A 200 µL solution of 50 mM sodium phosphate buffer at pH 8.0, containing 20 µM of GFP-tag-2, 2 mM cysteine, 2 mM TCEP.HCl, 1 mM FMeI, 20 µM SAH, 10 µM HMT and 10 µM $LahS_B$ was incubated at 25°C. After 1 min, 30 min, 100 min and 200 min, 10 µL aliquots of the reaction were quenched with 5 µL of 10 % formic acid. These solutions were submitted for analysis by HR-ESI-MS. These results show that $LahS_B$ F-methylated GFP-tag-3 efficiently, but no detectable ligation product (GFP-tag-3-Cysteine) formed.

Ligation between GFP-tag-1 and *O*-(4-nitrobenzyl) hydroxylamine

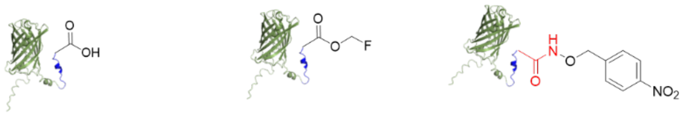

GFP-tag-1 30495.7 Da | **18** 30527.8 Da | **20** 30648.1 Da

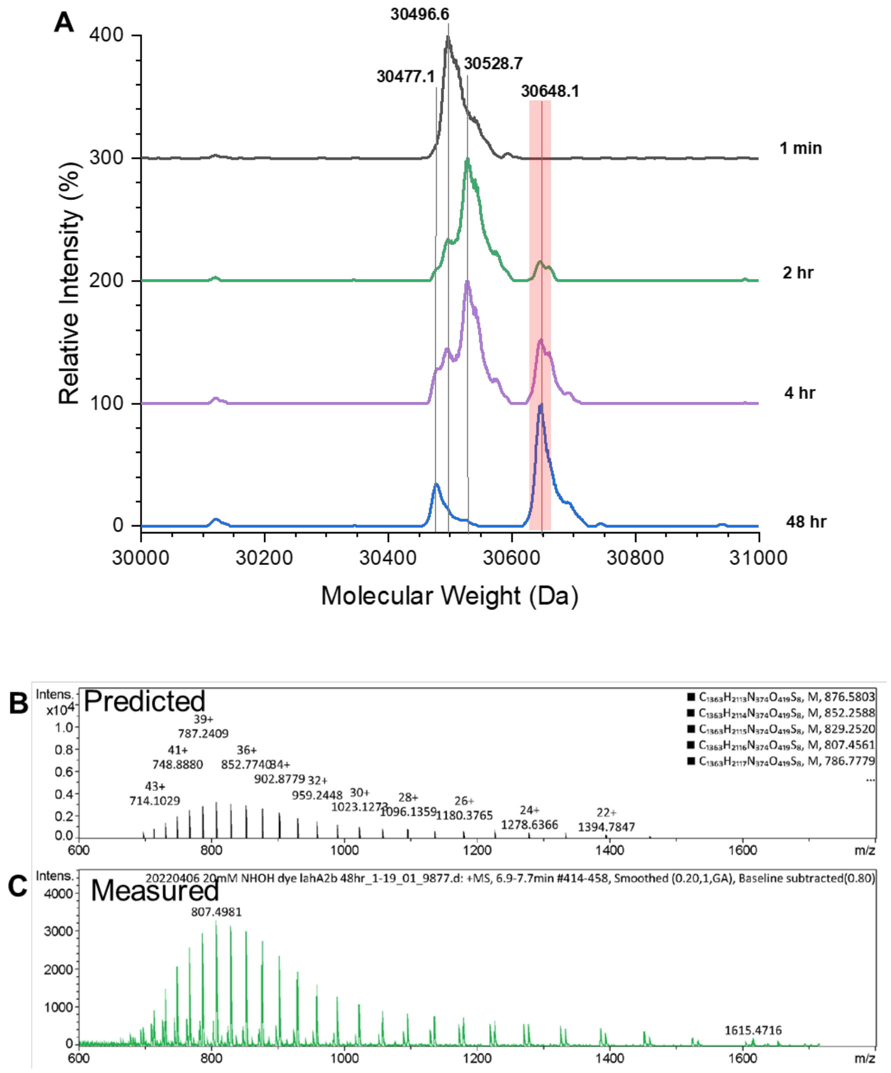

Figure S32. HR-ESI-MS spectra showing the ligation between GFP-tag-1 and *O*-(4-nitrobenzyl) hydroxylamine. **A:** deconvoluted mass spectra of the ligation reaction between GFP-tag-1 and *O*-(4-nitrobenzyl) hydroxylamine after 1 min, 2, 4, 48 hr. Comparison of the signal intensities suggest that approximately 70 % of GFP-tag-1 was ligated to *O*-(4-nitrobenzyl) hydroxylamine after 48 hr. **B:** predicted spectrum of conjugate **20**. **C:** measured spectrum of the reaction after 48 hr. The samples for this measurement were prepared as follows: A 200 µL solution of 50 mM sodium phosphate buffer at pH 8.0, containing 20 µM of GFP-tag-1, 20 mM of *O*-(4-nitrobenzyl) hydroxylamine, 20 µM SAH, 10 µM HMT, 10 µM $LahS_B$, and 1 mM FMeI was incubated at 25 °C. After 1 min, 2 hr, 4 hr and 48 hr, reaction aliquots were quenched with 10% formic acid. These solutions were submitted for analysis by HR-ESI-MS.

Ligation of GFP-tag-1 with biotin

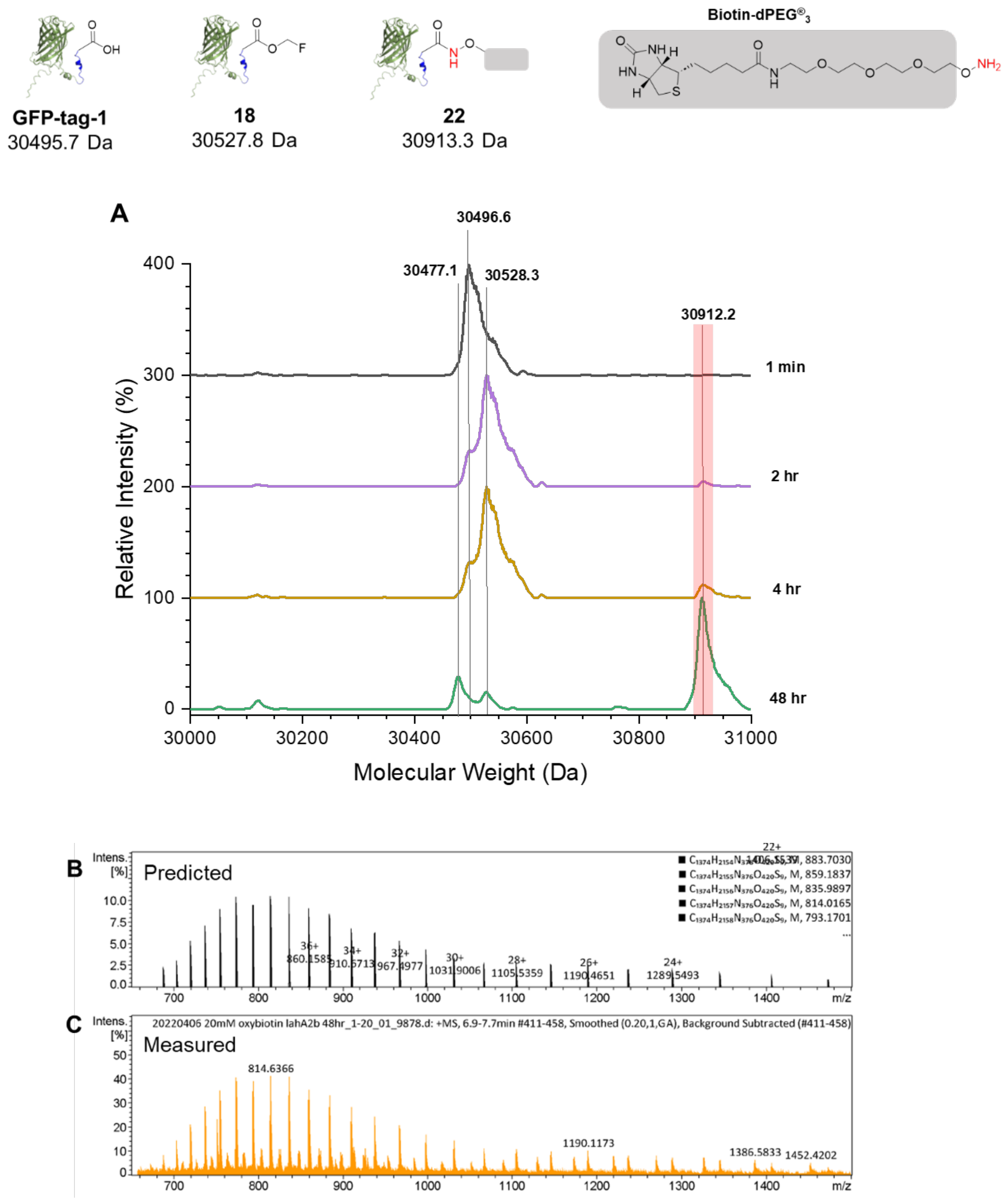

Figure S33. HR-ESI-MS spectra showing the ligation between GFP-tag-1 and biotin-dPEG®$_3$ oxyamine. **A:** deconvoluted mass spectra of the ligation reaction after 1 min, 2, 4, 48 hr. Comparison of the signal intensities suggest that approximately 70 % of GFP-tag-1 was ligated to biotin after 48 hr. **B:** predicted spectrum of compound **22**. **C:** measured spectrum of the reaction after 48 hr. The samples for this measurement were prepared as follows: A 200 µL solution of 50 mM sodium phosphate buffer at pH 8.0, containing 20 µM of GFP-tag-1, 20 mM of biotin-dPEG®$_3$-oxyamine, 20 µM SAH, 10 µM HMT, 10 µM LahS$_B$, and 1 mM FMeI was incubated at 25°C. After 1 min, 2 hr, 4 hr, and 48 hr, 1 µL aliquots were quenched by the addition of 10% formic acid. These solutions were submitted for analysis by HR-ESI-MS.

Ligation of GFP-tag-1 with hydrazine and chloroacetic acid

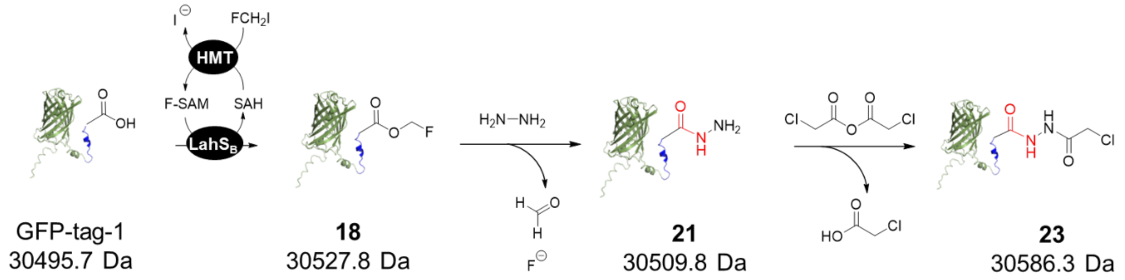

Scheme S10.

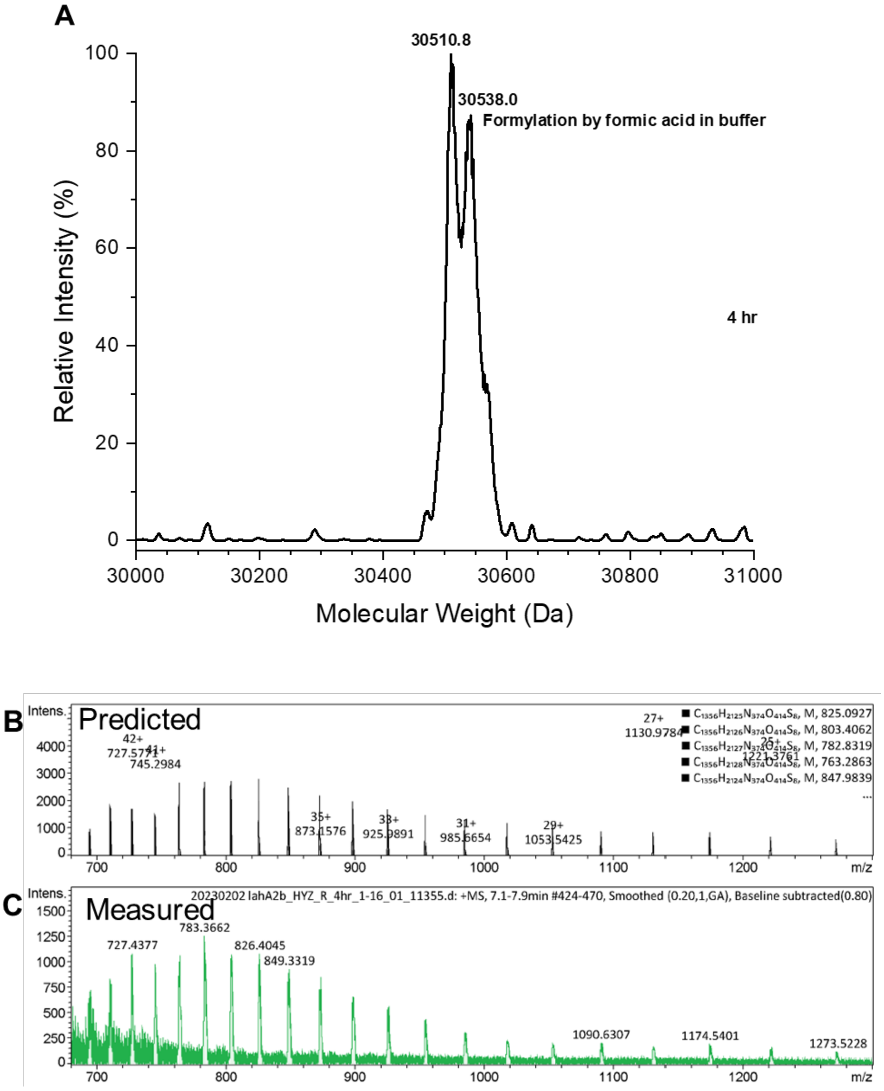

Figure S34. HR-ESI-MS spectra showing the ligation between GFP-tag-1 and hydrazine**. A:** deconvoluted mass spectra of the ligation reaction between GFP-tag-1 and hydrazine after 4 hr. This spectrum suggests that > 90 % of GFP-tag-1 was converted to compound **21** and a formylated derivative thereof. **B:** predicted spectrum of compound **21**. **C:** measured spectrum of the reaction after 4 hr. The samples for this measurement were prepared as follows: A 100 µL solution of 50 mM sodium phosphate buffer at pH 7.8 containing 100 µM GFP-tag-1, 40 µM SAH, 20 µM HMT, 20 µM LahS$_B$, 25 mM FMeI, and 20 mM hydrazine was incubated at 25°C. Additional doses of 400 µM FMeI were added after 1 and 2 hr. After 4 hours a 1 µL reaction aliquots were quenched by the addition of 1 µL of 10% formic acid. This solution was submitted for analysis by HR-ESI-MS.

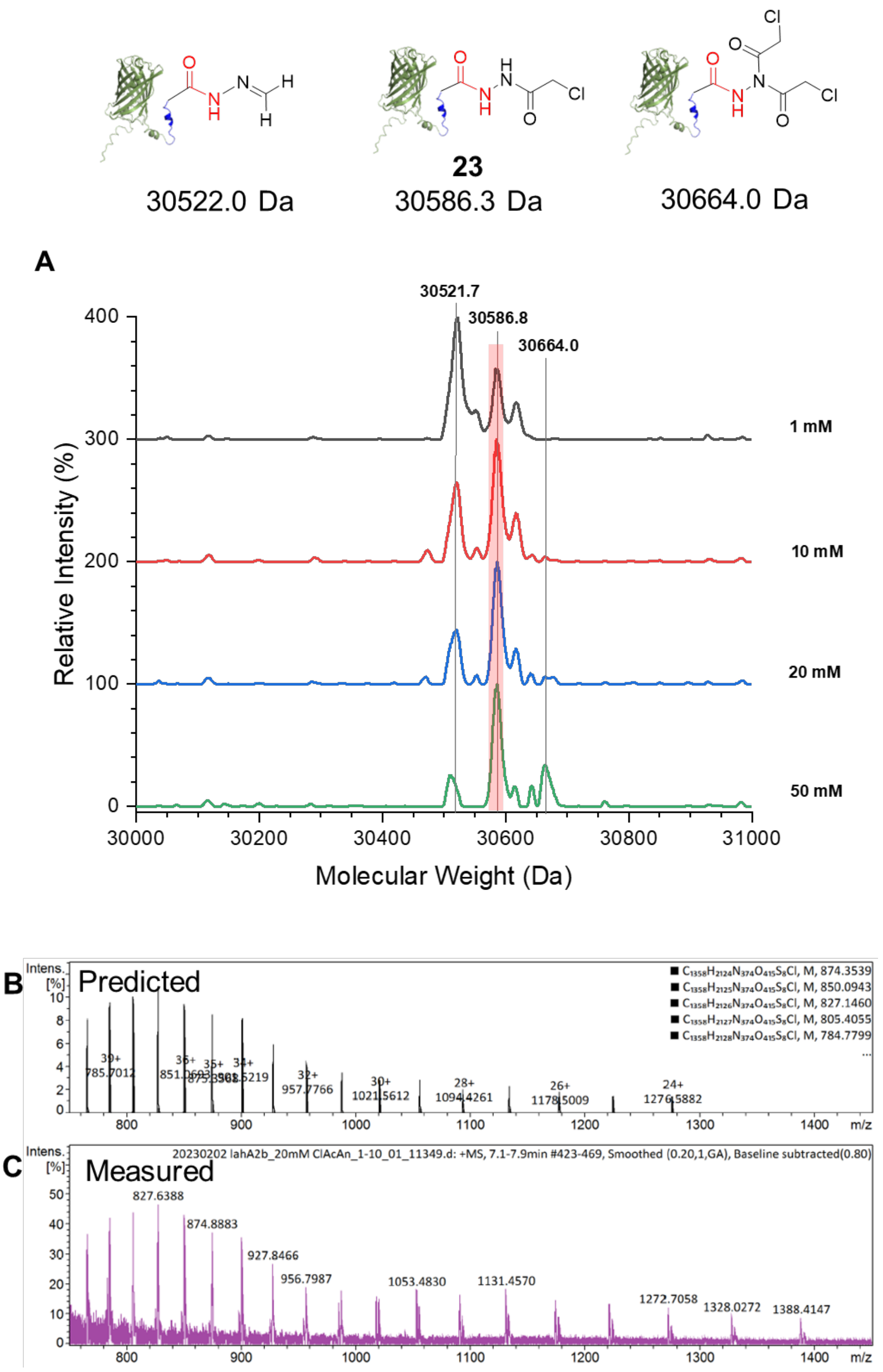

Figure S35. HR-ESI-MS spectra showing the ligation between **21** and chloroacetic anhydride. **A:** deconvoluted mass spectra of the ligation reaction between **21** and 1 mM, 10 mM, 20 mM or 50 mM of chloroacetic anhydride. These results show that incubation with 50 mM chloroacetic anhydride for 10 min convert 72% of **21** to conjugate **23**. **B:** predicted spectrum of compound **23**. **C:** measured spectrum of the reaction incubated with 20 mM chloroacetic anhydride. The samples for this measurement were prepared as follows: 50 µL of the **21**-containing reaction solution described in Figure S34 was diluted to 500 µL in 50 mM NaH_2PO_4 buffer, at pH 3.0. 100 µL aliquots of this solution were supplemented with 1 mM, 10 mM, 20 mM or 50 mM chloroacetic anhydride from a 500 mM stock solution in DMF. The resulting mixtures were incubated for 10 min at 25 °C. These solutions were submitted for analysis by HR-ESI-MS. The procedure to convert **21** to **23** was inspired from a published protocol.[5] Two additional species were tentatively identified as the hydrazone (calcd: m/z 30522.0, found: 30522) forming by condensation between **21** and formaldehyde; and a species emerging from the attachment of a second 2-chloroacetyl group to **23** (calcd: m/z 30664.0, found: 30664).

Auto-Ligation

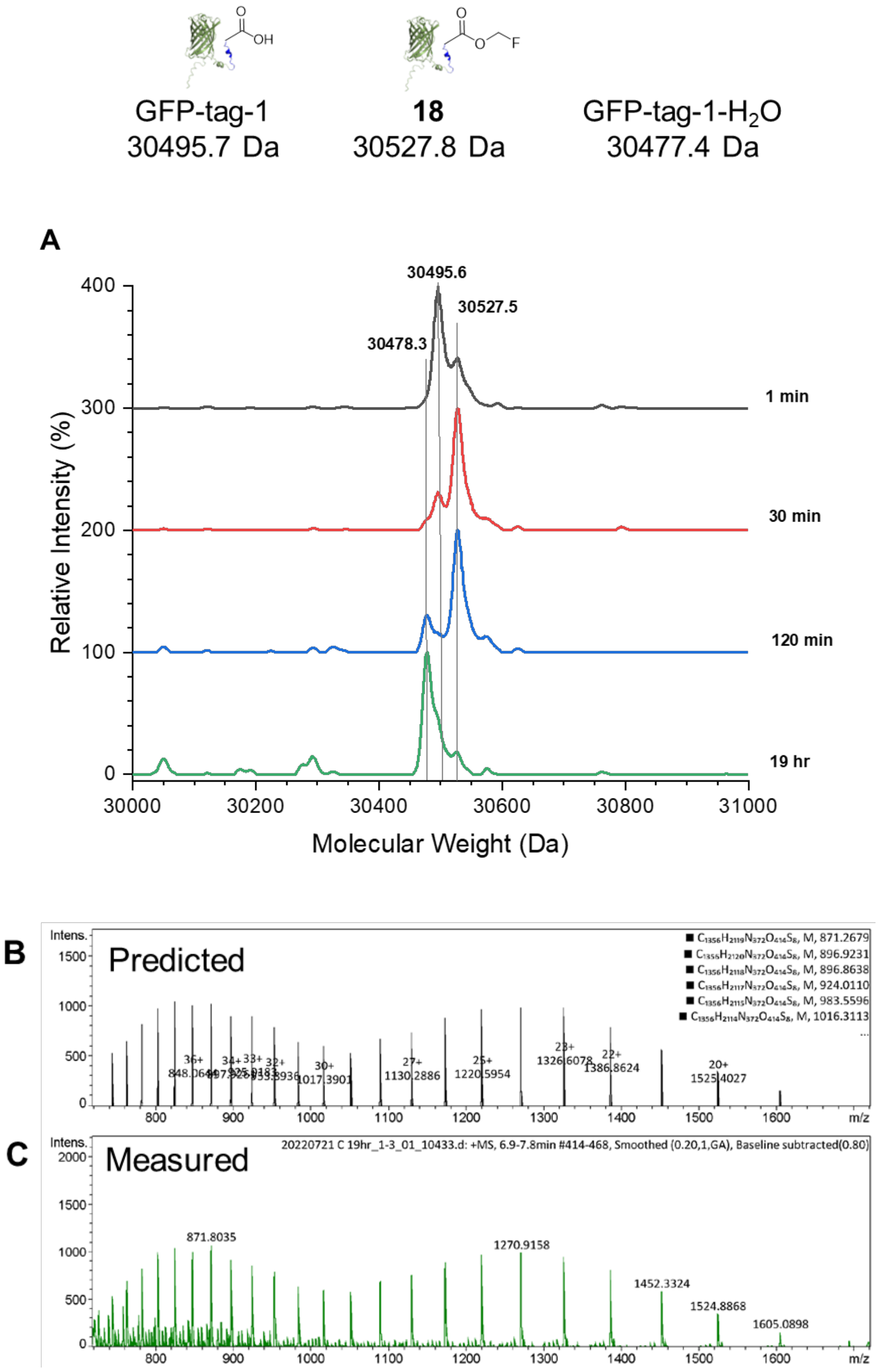

Figure S36. HR-ESI-MS spectra showing the auto-ligation F-methylated GFP-tag-1 (**18**). **A:** deconvoluted mass spectra of the ligation reaction after 1, 30, 120 min and 19 hr. These spectra show that GFP-tag-1 (> 80 %) slowly reacts to an anhydride (GFP-tag-1-H_2O) after being F-methylated in the absence of a strong nucleophile such as cysteine. **B:** predicted spectrum of aute-ligation product of GFP-tag-1-H_2O. **C:** measured spectrum of 19 hr reaction. The samples for this measurement were prepared as follows: A 200 µL solution of 50 mM sodium phosphate buffer at pH 8.0, containing 20 µM GFP-tag-1, 1 mM FMeI, 20 µM SAH, 10 µM HMT and 10 µM $LahS_B$ was incubated at 25°C. After 1, 30, 120 min and 19 hr, 10 µL reaction aliquots were quenched by the addition of 5 µL of 10% formic acid. These solutions were submitted for analysis by HR-ESI-MS.

Ligation between nanobody and cysteine

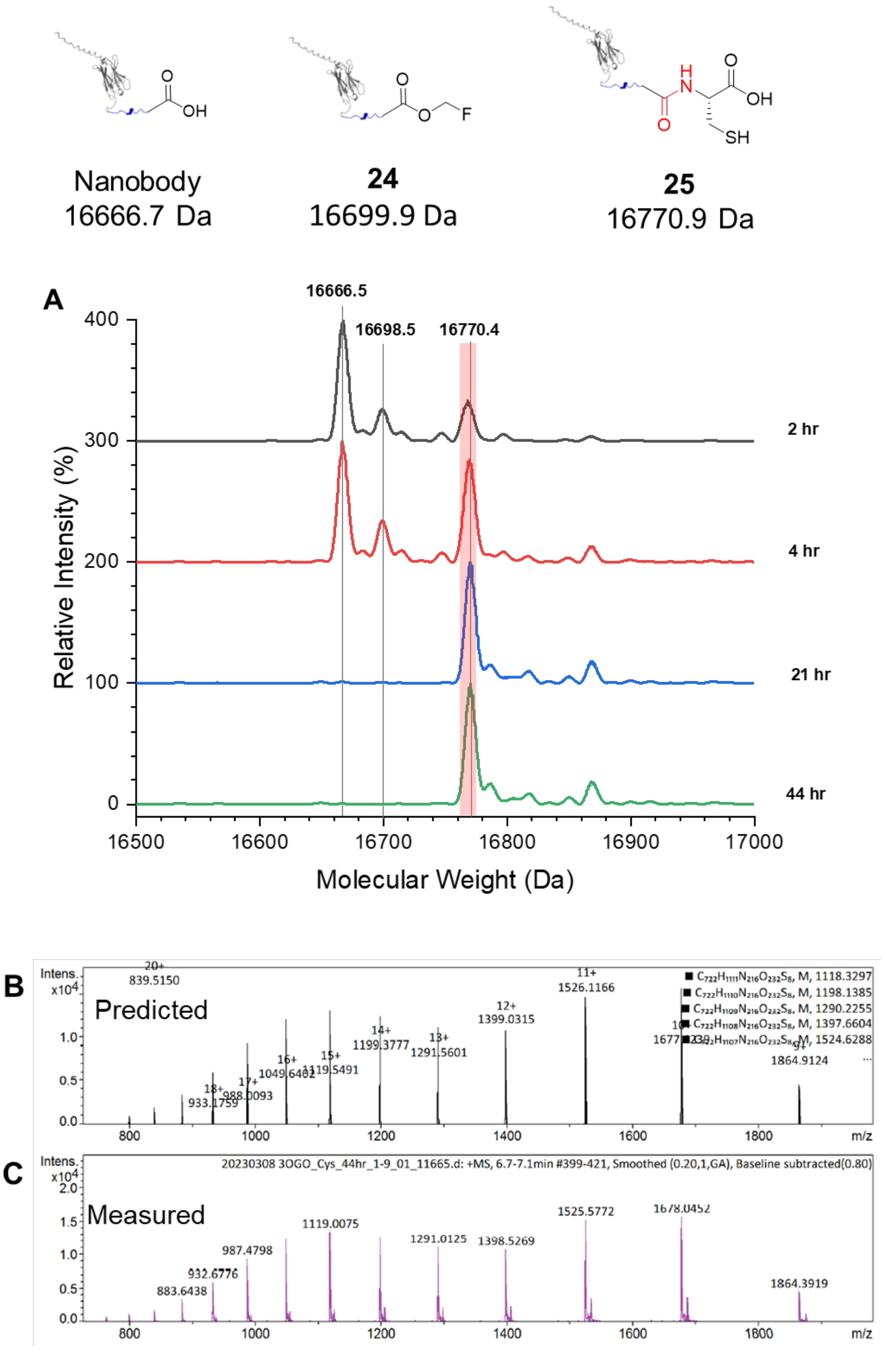

Figure S37. HR-ESI-MS spectra showing the ligation between nanobody and cysteine. **A:** deconvoluted mass spectra of the ligation reaction after 2, 4, 21, and 44 hr. Comparison of the signal intensities suggest that > 90 % of the nanobody was converted to the conjugate **25**. **B:** predicted spectrum of compound **25**. **C:** measured spectrum of the reaction after 44 hours. The samples for this measurement were prepared as follows: A 200 µL solution of 50 mM sodium phosphate buffer at pH 8.0, containing 100 µM of nanobody, 3 mM cysteine, 3 mM TCEP, 20 µM SAH, 10 µM HMT, and 10 µM $LahS_B$ was incubated at 15°C. Additional doses of 400 µM of FMeI were added after 4, 19, 22 hour. After 2, 4, 21 and 44 hr, 10 µL reaction aliquots were quenched by the addition of 5 µL of 10 % formic acid. These solutions were submitted for analysis by HR-ESI-MS.

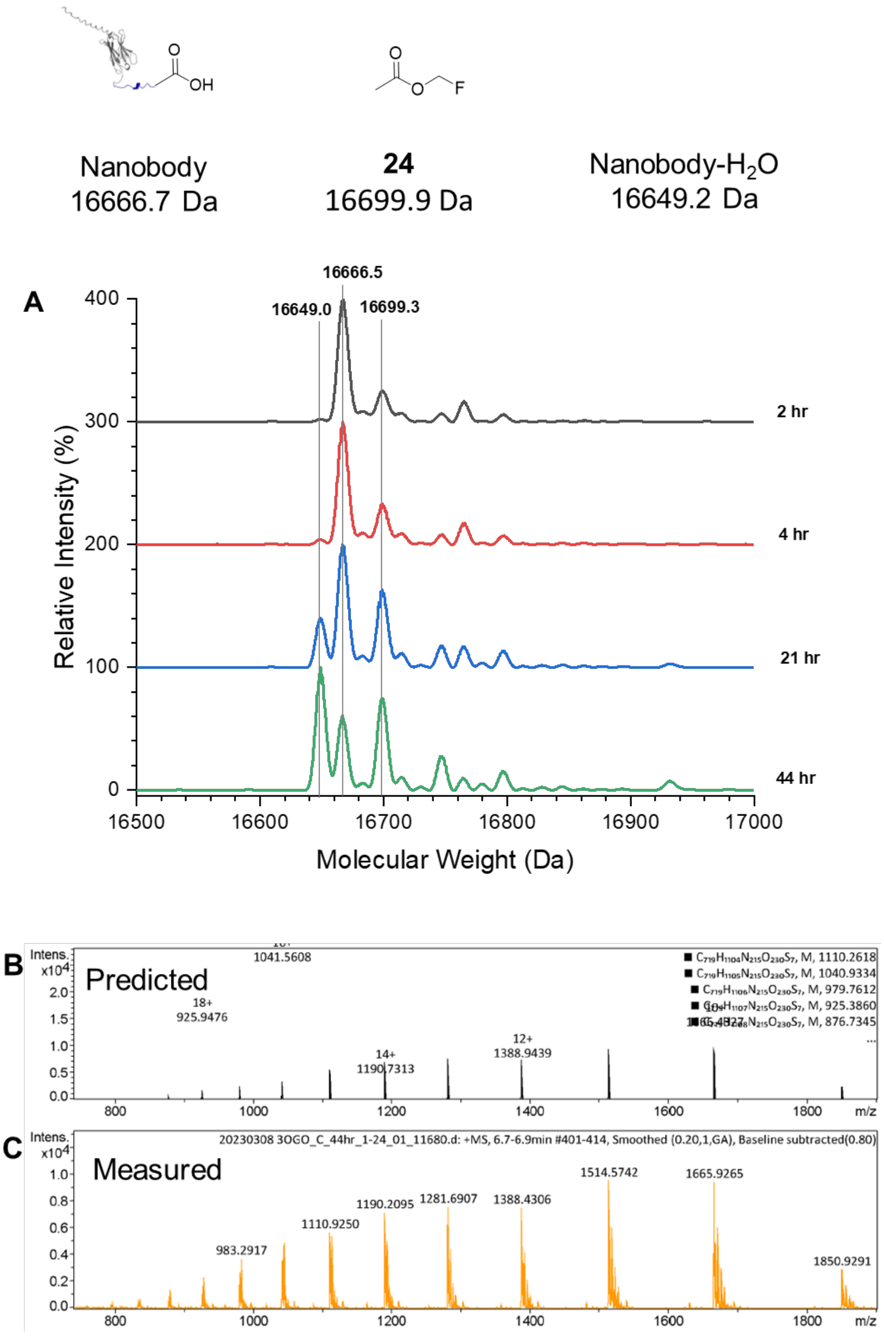

Figure S38. HR-ESI-MS spectra showing the auto-ligation of nanobody in the absence of cysteine. **A:** deconvoluted mass spectra of the ligation reaction at 2 hr, 4 hr, 21 hr, 44 hr show that up to 40 % of nanobody was converted to an anhydride (nanobody-H_2O) after being F-methylated in the absence of a strong nucleophile such as cysteine. **B:** predicted spectrum of nanobody-H_2O. **C:** measured spectrum of the reaction after 44 hours. The samples for this measurement were prepared as follows: A 20 µL solution of 50 mM sodium phosphate buffer at pH 8.0, containing 100 µM nanobody, 3 mM TCEP, 4 mM FMeI, 20 µM SAH, 10 µM HMT and 10 µM LahS$_B$ was incubated at 15°C. Additional doses of 400 µM of FMeI were added after 4, 19, 22 hr. After 2, 4, 21 and 44 hr, 10 µL reaction aliquots were quenched by the addition of 5 µL of 10% formic acid. These solutions were submitted for analysis by HR-ESI-MS.

Ligation between nanobody and *O*-(4-nitrobenzyl) hydroxylamine

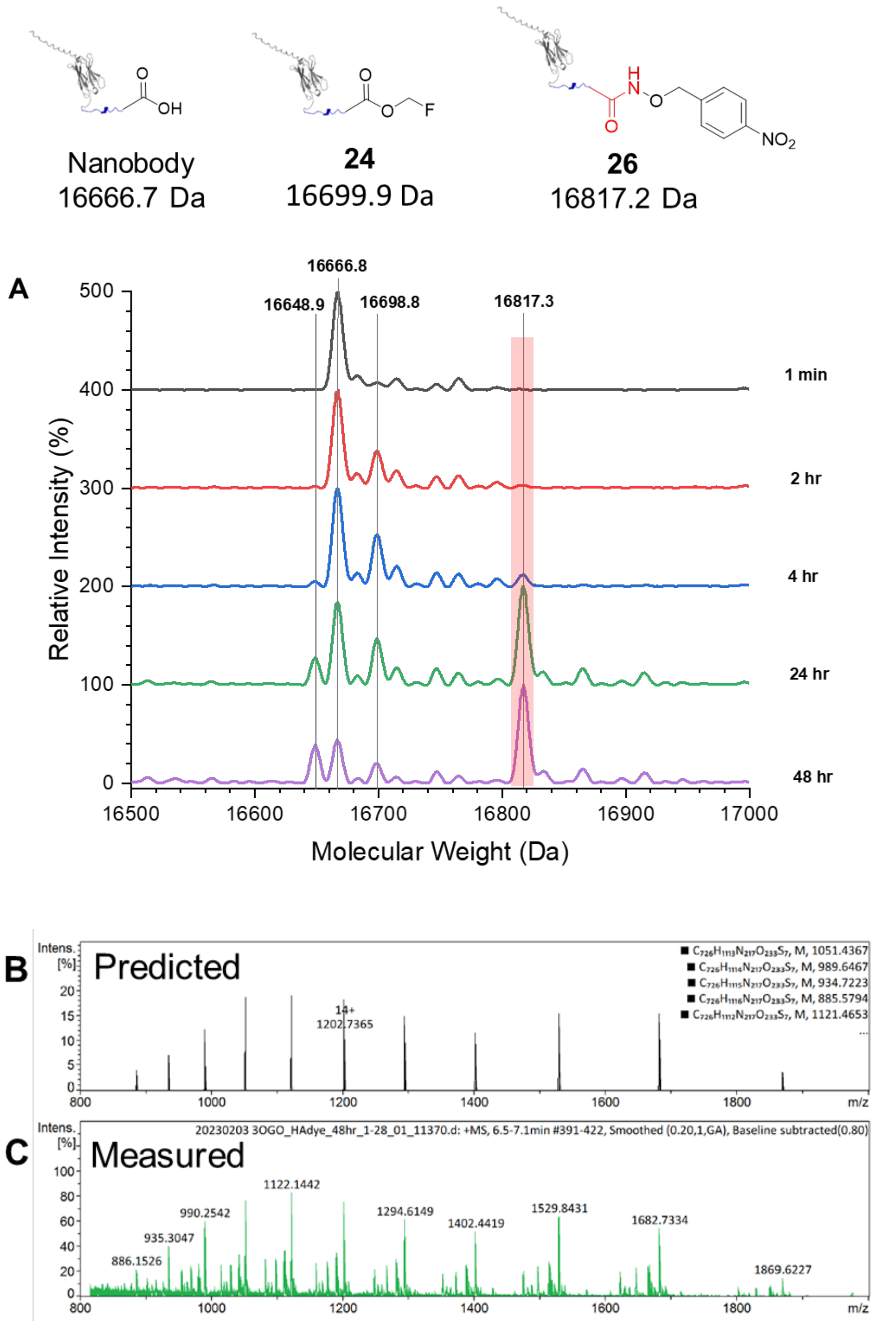

Figure S39. HR-ESI-MS spectra showing the ligation between nanobody and *O*-(4-nitrobenzyl) hydroxylamine. **A:** deconvoluted mass spectra of the reaction at 1 min, 2 hr, 4 hr, 24 hr, 48 hr. Comparison of the signal intensities suggest that approximately 70 % of nanobody was converted to the ligation product **26**. **B:** predicted spectrum of **26**. **C:** measured spectrum of the reaction after 48 hours. The samples for this measurement were prepared as follows: A 20 µL solution of 50 mM sodium phosphate buffer at pH 8.0, containing 100 µM of nanobody, 2 mM *O*-(4-nitrobenzyl) hydroxylamine, 10 µM HMT, 10 µM LahS$_B$, 4 mM FMeI, and 20 µM SAH was incubated at 25°C. After 1 min, 2, 4, 24, 48 hr reaction aliquots were quenched by the addition of 10% formic acid. These solutions were submitted for analysis by HR-ESI-MS.

Ligation between nanobody and biotin-dPEG®$_3$ oxyamine

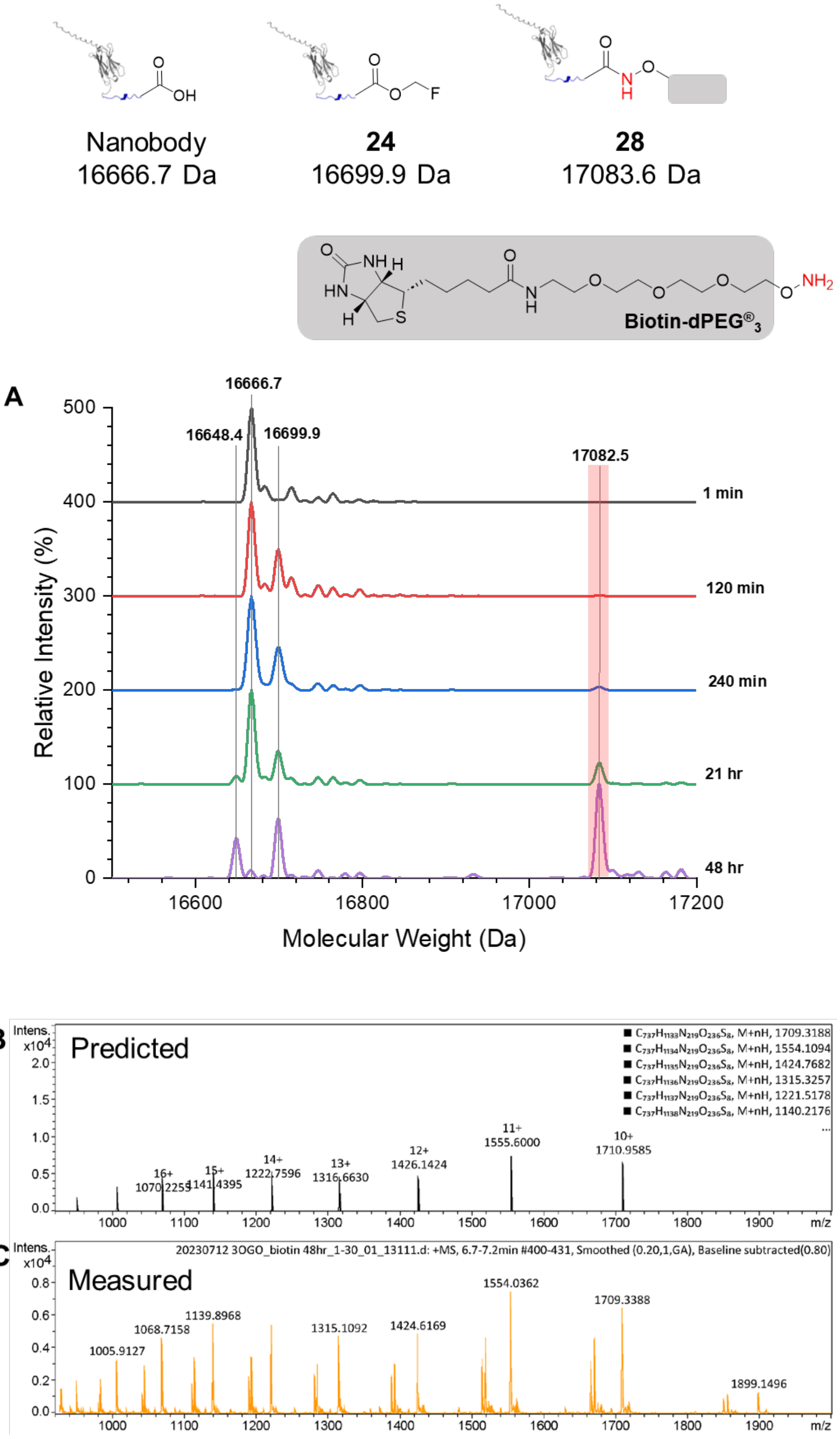

Figure S40. HR-ESI-MS spectra showing the ligation between nanobody and biotin-dPEG®$_3$ oxyamine. **A:** deconvoluted mass spectra of the ligation reaction between nanobody and biotin after 1 min, 2, 4 and 21 hr and 48 hr reaction. Comparison of the signal intensities suggest that approximately 50% of nanobody was converted to compound **28** after 48 hr. **B:** predicted spectrum of compound **28**. **C:** measured spectrum of the reaction after 48 hr. The samples for this measurement were prepared as follows: A 20 µL solution of 50 mM sodium phosphate buffer at pH 7.8, containing 50 µM of nanobody, 20 µM HMT, 20 µM LahS$_B$, 40 µM SAH, 4 mM FMeI, and 40 mM biotin-dPEG®$_3$ oxyamine was incubated at 25°C. Additional doses of 400 µM of FMeI were added after 1 and 2 hr. After 1 min, 2, 4 and 21 hr, 1 µL aliquots were quenched with 10% formic acid. These solutions were submitted for analysis by HR-ESI-MS.

Ligation between nanobody and hydrazine

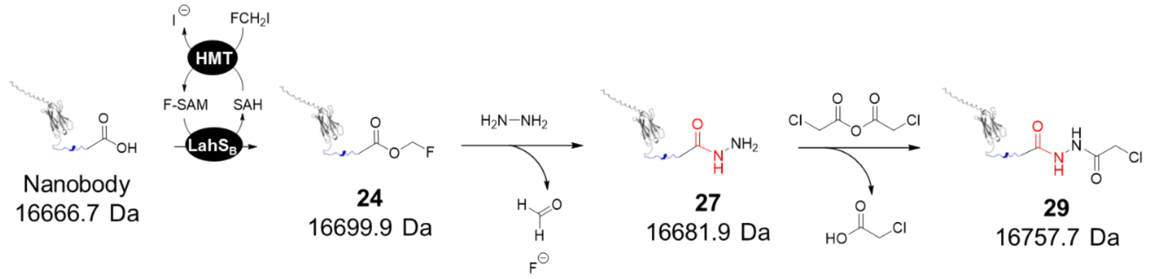

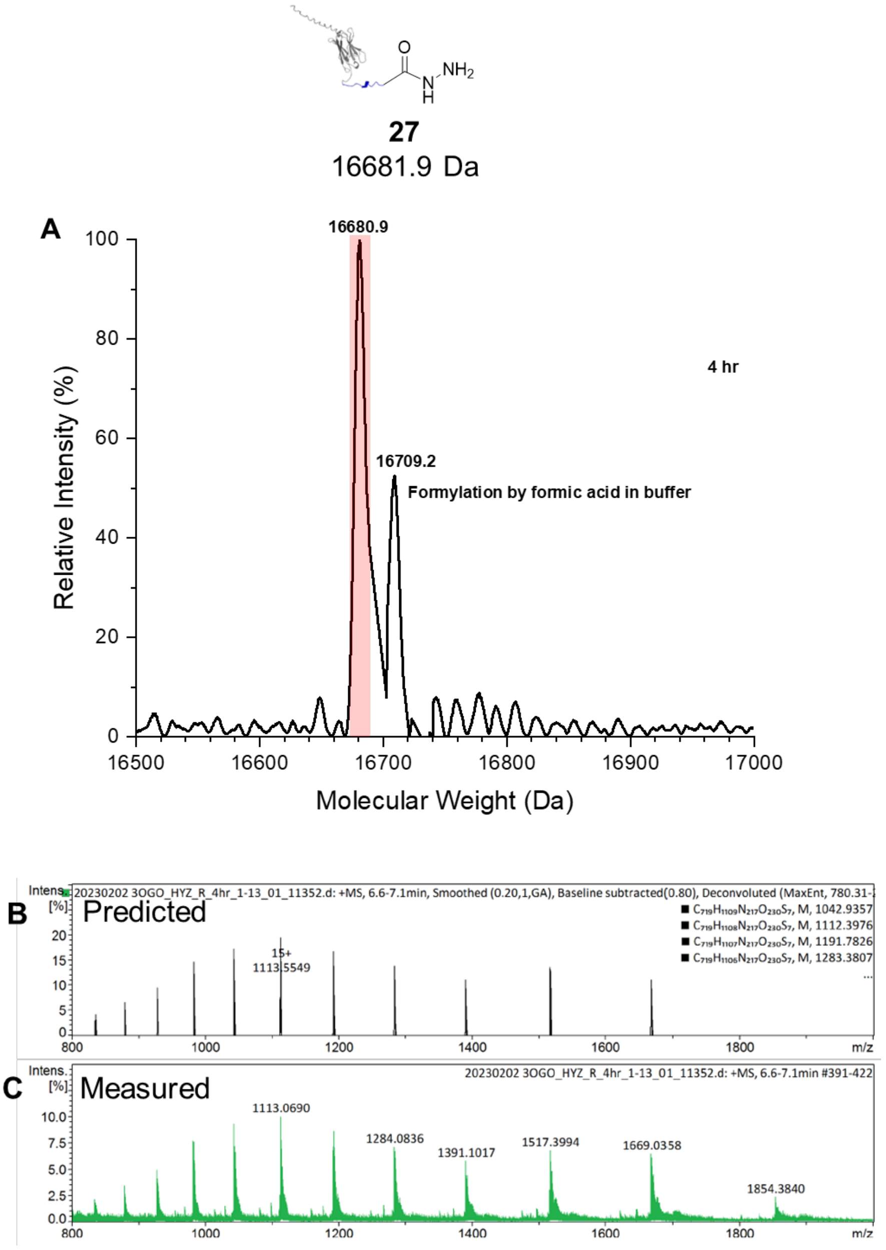

Figure S41. HR-ESI-MS spectra showing the ligation between nanobody and hydrazine. **A:** deconvoluted mass spectra of the ligation reaction between nanobody and hydrazine after 4 hr reaction. This spectrum suggests that > 90 % of nanobody was converted to compound **27** and a formylated derivative thereof. **B:** predicted spectrum of compound **27**. **C** measured spectrum of the reaction after 4 hr. The samples for this measurement were prepared as follows: A 20 µL solution of 50 mM sodium phosphate buffer at pH 7.8 containing 100 µM nanobody, 20 µM HMT, 20 µM LahS$_B$, 40 µM SAH, 5 mM FMeI, 20 mM hydrazine. Additional doses of 400 µM of FMeI were added after 1 and 2 hr. After 4 hours a 1 µL reaction aliquots was quenched by the addition of 1 µL of 10% formic acid. This solution was submitted for analysis by HR-ESI-MS.

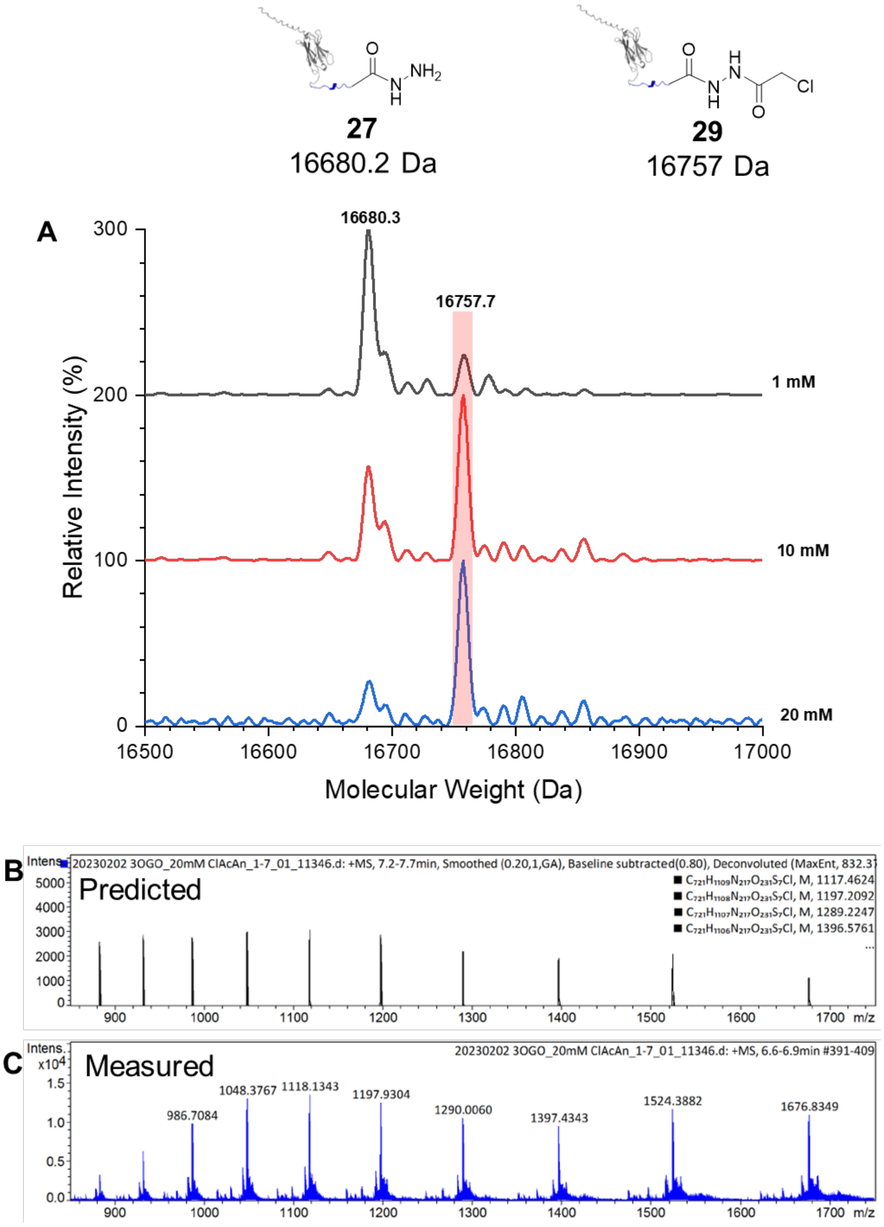

Figure S42. HR-ESI-MS spectra showing the ligation between **27** and chloroacetic anhydride. **A:** deconvoluted mass spectra of the ligation reaction between **21** and 1 mM, 10 mM, or 20 mM chloroacetic anhydride. These results show that incubation with 20 mM chloroacetic anhydride for 10 min convert 80 % of **27** to conjugate **29**. B**:** predicted spectrum of compound **29**. **C:** measured spectrum of the reaction incubated with 20 mM of chloroacetic anhydride. The samples for this measurement were prepared as follows: 50 µL of the **27**-containing reaction solution described in Figure S41 was diluted to 500 µL in 50 mM NaH_2PO_4 buffer, at pH 3.0. 100 µL aliquots of this solution were supplemented with 1 mM, 10 mM or 20 mM chloroacetic anhydride from a 500 mM stock solution in DMF. The resulting mixtures were incubated for 10 min at 25 °C. These solutions were submitted for analysis by HR-ESI-MS. The procedure to convert **27** to **29** was inspired from a published protocol.[5]

Protein-protein ligation

In early attempts to ligate GFP-tag-1 to nanobody-C16 we observed the rapid formation of a GFP derivative with a 11 Da higher mass that the parent protein. We surmised that this species occurs because the N-terminal Cys residue reacts with formaldehyde to produce a thiazolidine, which in turn is a degradation product of FMeI. To suppress this passivating side reaction, we included 5 uM of a glutathione-independent formaldehyde dehydrogenase from *Pseudomonas putida* (EC:1.2.1.46) in the ligation reaction. This NAD^+-dependent enzyme catalyzes redox neutral dismutation of two equivalents of formaldehydes to methanol and formic acid.[6]

In 20 μL of reaction in 50 mM sodium phosphate buffer at pH 8.0, 15 °C, 0.3 mM of GFP-tag-1 and 0.6 mM of nanobody-C16 were incubated with 20 μM HMT, 20 μM $LahS_B$, 40 μM SAH, 10 μM FalD (formaldehyde dismutase), 0.2 mM NAD, 0.2 mM $ZnCl_2$, 4 mM FMeI, 2 μM TEV protease. In the control reaction, $LahS_B$, or HMT, or nanobody-C16, or HMT and $LahS_B$ was replaced with equal volume of sodium phosphate buffer. 1 mM of FMeI was added at 3, 25 hr. 2 mM of FMeI was added after 22 hr of incubation. After 48 hours of reaction, 1 μL of the reaction was mixed with 10 mM DTT, SDS loading buffer (240 mM Tris-HCl, 6% SDS, 30% glycerol, 0.9 mM Bromophenolblue) and water. The total sample volume was 20 μL. The SDS-PAGE samples were heated at 95 °C for 5 min and centrifuged at 9,000 rcf for 1 min. The reaction mixtures were diluted and submitted for analysis by HR-ESI-MS. The expected protein sequence is as follow, the connectivity between two proteins is highlighted in red. The N-terminal His-tag has been removed from all proteins involved.

```
>GFP-tag-1-Nanobody-C16 (N-terminal His tag eliminated) Molecular Weight: 41782.1
GSGMSKGEELFTGVVPILVELDGDVNGHKFSVRGEGEGDATNGKLTLKFICTTGKLPVPWPTLVTTLTYGVQCFSRYPDHMKRHDFFKSAMPEG
YVQERTISFKDDGTYKTRAEVKFEGDTLVNRIELKGIDFKEDGNILGHKLEYNFNSHNVYITADKQKNGIKANFKIRHNVEDGSVQLADHYQQN
TPIGDGPVLLPDNHYLSTQSVLSKDPNEKRDHMVLLEFVTAAGITHGMDELYKGSGDGDEVDYSLFAATAMCSGSQVQLVESGGALVQPGGSLR
LSCAASGFPVNRYSMRWYRQAPGKEREWVAGMSSAGDRSSYEDSVKGRFTISRDDARNTVYLQMNSLKPEDTAVYYCNVNVGFEYWGQGTQVTV
SS

>GFP-tag-1 (N-terminal His tag eliminated) Molecular Weight: 28750.3
GSGMSKGEELFTGVVPILVELDGDVNGHKFSVRGEGEGDATNGKLTLKFICTTGKLPVPWPTLVTTLTYGVQCFSRYPDHMKRHDFFKSAMPEG
YVQERTISFKDDGTYKTRAEVKFEGDTLVNRIELKGIDFKEDGNILGHKLEYNFNSHNVYITADKQKNGIKANFKIRHNVEDGSVQLADHYQQN
TPIGDGPVLLPDNHYLSTQSVLSKDPNEKRDHMVLLEFVTAAGITHGMDELYKGSGDGDEVDYSLFAATAM

>GFP-tag-1-H2O (N-terminal His tag eliminated) Molecular Weight: 28732.3

>Nanobody-C16 (N-terminal His tag eliminated) Molecular Weight: 13050.5
CSGSQVQLVESGGALVQPGGSLRLSCAASGFPVNRYSMRWYRQAPGKEREWVAGMSSAGDRSSYEDSVKGRFTISRDDARNTVYLQMNSLKPED
TAVYYCNVNVGFEYWGQGTQVTVSS

>LahSB (N-terminal His tag eliminated) Molecular Weight: 35396.4
GSGMEKEIKKWSVYFQNPEFLERTRMFLIQKELYPLVRNWCGVKDNVRLLDVGCGTGYFTRLLVSGDEDVSAVGIDMEEPFIEYAREKAEELGL
PAEFIIGDALALPFEDNTFDIVTSHTFLTSVPDPEKAMSEMKRVVKPGGIISSVTAMNFMPACNNEGEYPEECTWVEDLKKEYMKIYTKYFSAD
PLETRIKGVKCSDVPKFFTGQGLKDVSLYPIGKVFTLSNAAVSDEDKLRYIELFYASEIKKLDAFMELPDEDIGITEEDAERFRSLIGQKCKWL
RDHLHDNYAWEWQGGANLLVTGICNKQR

>BxHMT (N-terminal His tag eliminated) Molecular Weight: 22919.7
GSGSDPTQPAVPDFETRDPNSPAFWDERFERRFTPWDQAGVPAAFQSFAARHSGAAVLIPGCGSAYEAVWLAGQGNPVRAIDFSPAAVAAAHEQ
LGAQHAQLVEQADFFTYEPPFTPAWIYERAFLCALPLARRADYAHRMADLLPGGALLAGFFFLGATPKGPPFGIERAELDALLTPYFDLIEDEA
VHDSIAVFAGRERWLTWRRRA
```

Electrophoretic analysis. 10% Tris-glycine SDS-polyacrylamide resolving gel (H_2O, 37.5:1% acrylamide/bisacrylamide mix, 1 M Tris buffer pH 8.8, 20% SDS, 20% ammonium persulfate and TEMED) and 5% stacking gel (H_2O, 37.5:1% acrylamide/bisacrylamide mix, 1.0 M Tris buffer pH 6.8, 20% SDS, 20% ammonium persulfate, TEMED) were packed between Mini-PROTEAN® Short Plates. 10× running buffer stock (250 mM Tris base, 2 M glycine, 1% w/v SDS in deionised water) was diluted into 1× running buffer. 700 mL of the 1× running buffer was added to the inner chamber and the tank. Individual gel lanes were loaded with protein ladder standard (1.5 μL), protein starting material (GFP-tag-1 and nanobody C16), and control reactions, or sample (5 μL each). Electrophoretic separations were run at 50 V for 20 min, to pre-pack the samples, and then at 120 V for 60 min. The gel was stained with QuickBlue Protein Stain (lubio science) for 40 min and then de-stained in deionized water over 2 days at room temperature. The gel was photographed using BIO-RAD GelDoc Go Imaging System and stored wet in plastic wallets at 4 °C.

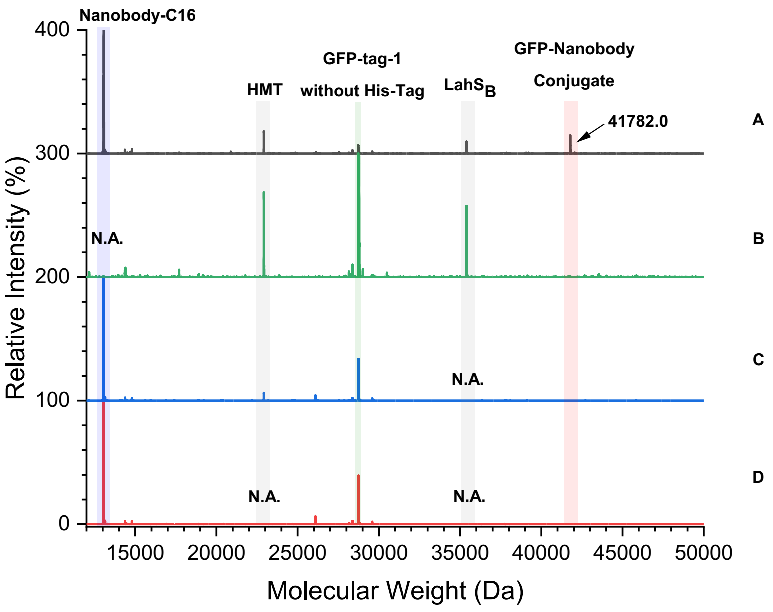

Figure S43. HR-ESI-MS spectra showing the ligation between GFP-tag-1 and nanobody-C16. **A:** reaction, the black indicates the ligation product GFP-nanobody with a molecular weight of 41782.0. **B:** control reaction lacking nanobody-C16. **C:** control reaction lacking LahS$_B$. **D:** control reaction lacking HMT and LahS$_B$.

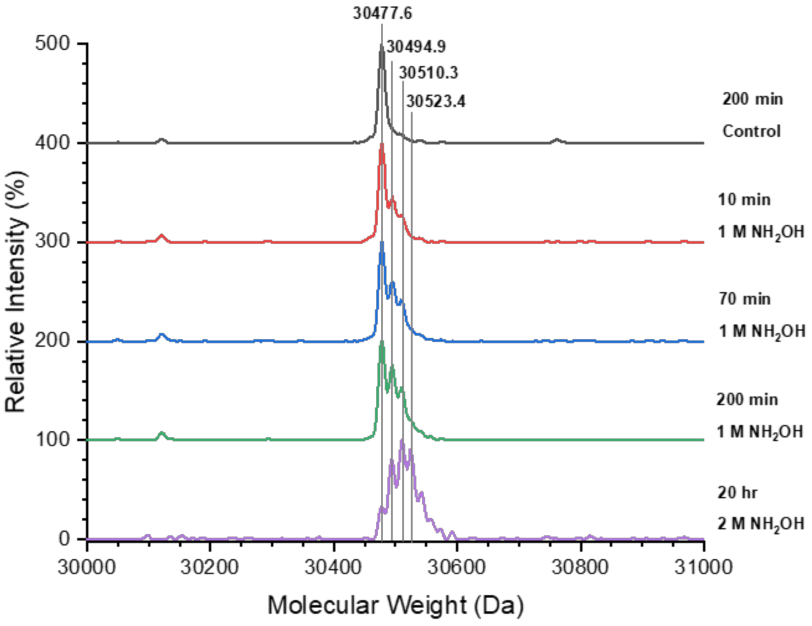

Figure S44. HR-ESI-MS spectra showing that the anhydride species GFP-tag-1-H_2O undergoes hydrolysis or hydroxylaminolysis upon incubation with NH_2OH. To generate GFP-tag-1-H_2O (m/z calc. 30477.3), we incubated 50 µM GFP-tag-1 with 1 mM of FMeI, 20 µM HMT, 20 µM LahS$_B$, 40 µM SAH in 50 mM sodium phosphate buffer at pH 8.0, 25 °C overnight. 10 µL of the reaction mixture was mixed with 10 µL of a 2 M NH_2OH solution that was adjusted to pH 8.0. 0.5 µL of the mixture was diluted and analyzed by HRMS after 10, 70, 200 min. A control reaction was incubated at pH 8.0 buffer for 200 min (control). Overnight incubation (20 h) in the presence of 2 M of NH_2OH lead to almost complete cleavage of GFP-tag-1-H_2O to the hydrolysis product GFP-tag-1 (m/z 30494.9) or the aminolysis product GFP-tag-1-hydroxamate (m/z 30510.3).

Reference

[1] C. Liao, F. P. Seebeck, *Nat. Catal.* **2019**, *2*, 696–701.

[2] J. D. Pédelacq, S. Cabantous, T. Tran, T. C. Terwilliger, G. S. Waldo, *Nat. Biotechnol.* **2006**, *24*, 79–88.

[3] J. Peng, C. Liao, C. Bauer, F. P. Seebeck, *Angew. Chemie* **2021**, *133*, 27384–27389.

[4] T. Zhang, K. Hansen, A. Politis, M. M. Müller, *Biochemistry* **2020**, *59*, 3683–3695.

[5] J. Farnung, K. A. Tolmachova, J. W. Bode, *Chem. Sci.* **2022**, *14*, 121–129.

[6] N. Tanaka, Y. Kusakabe, K. Ito, T. Yoshimoto, K. T. Nakamura, *J. Mol. Biol.* **2002**, *324*, 519 - 533.
